# Supplementary material for: Amphibious microneedles for programmable delivery of biomolecules and microorganisms in living plants
Source: Nat Commun. 2025 Nov 24;16:10993. doi: 10.1038/s41467-025-66799-1 (PMC12690088; doi:10.1038/s41467-025-66799-1)
Supplement: Supplementary file 1 — Supplementary Information [file 41467_2025_66799_MOESM1_ESM.pdf]

Amphibious microneedles for programmable delivery of biomolecules and microorganisms in living plants

Shen *et al.*

Supplementary Method 1. Preparation of core-shell MNP with cargo in microneedle tips

The microneedle preparation process followed the mould-aiding strategy. Briefly, RhB (cargo), HA and $KHCO_3$ were dissolved in water to make Solution A (0.1% RhB + 8% HA + 2% $KHCO_3$). PCL and C10 were dissolved in DCM to make Solution B (7.5% PCL + 2.5% C10). 100 mg of Solution A was added into the microneedle mould. The solution-mould system was kept in a vacuum chamber (20 mbar) for 15 minutes. Then the system was moved back to room pressure and the solution in the back region was completely removed. After that, the system was kept in a desiccator for 4 hours, where water would evaporate and the RhB-loaded microneedle tips would form. HA/$KHCO_3$ solution (8% HA + 2% $KHCO_3$) was then added to the back region of the mould followed by 12-hour desiccation. After this treatment, the core MNP (with RhB only in microneedle tips) could be detached from the mould. This core MNP was subsequently immersed in Solution B for 5 seconds. After 12-hour evaporation of DCM, the core-shell MNP (with RhB only in microneedle tips) were obtained. Percentages are on a weight basis.

Supplementary Method 2. Preparation and characterization of hydrophobic MNP

For the blank hydrophobic MNP, 100 mg of 10wt% PCL was added into the microneedle mould. The solution-mould system was kept in a vacuum chamber (20 mbar) for 5 minutes and a desiccator for 12 hours, successively. After this treatment, the hydrophobic MNP was ready and could be detached from the mould.

To prepare the plasmid-loaded hydrophobic MNP, 1000 ng GFP-encoding plasmid DNA was mixed into 100 mg of 10wt% PCL before adding into the microneedle mould. The rest steps were the same as the preparation of blank hydrophobic MNP. The plasmid DNA was extracted from the MNP overnight for the further test. Agarose gel electrophoresis was then performed to analyze the fresh plasmid sample and that extracted from MNP. 2% (w/v) agarose gel, stained by FloroSafe DNA Stain (1st BASE), was used for the study. Electrophoresis was conducted at 100 V for 30 minutes. DNA bands were imaged by Amersham ImageQuant 800 under UV illumination.

To prepare the *A. tumefaciens*-loaded hydrophobic MNP, ~10^8 CFU of fresh *A. tumefaciens* was mixed into 100 mg of 10wt% PCL before adding into the microneedle mould. The rest steps were the same as the preparation of blank hydrophobic MNP. The *A. tumefaciens* was extracted from the MNP overnight for the further test. 100 μL of the extraction liquid was added and spread on the LB agar plate (containing 0.1 mg/mL gentamicin, 0.1 mg/mL kanamycin and 0.05 mg/mL rifampicin). The bacterial colonies were counted after 3-day incubation under 30 °C.

Supplementary Note 1. Optimization of MNP fabrication

Using HFIP as an alternative solvent to fabricate the core-shell MNP

As shown in Supplementary Fig. 6a, b, HFIP successfully replaced DCM for producing core-shell microneedles with comparable morphology and structural integrity. GC/MS analysis confirmed the absence of residual HFIP in the final microneedle product (Supplementary Fig. 6c). Moreover, we used *A. tumefaciens* as a model cargo. The recovered bacterial count reached $(1.31 \pm 0.14) \times 10^5$ CFU from one MNP, demonstrating that the HFIP-based strategy is compatible with the preservation of biological activity (Supplementary Fig. 6d).

Effect of viscosity on microneedle tip geometry

The geometry of the core-shell microneedles is mainly influenced by the viscosity of the coating solution, which forms the outer shell (Supplementary Fig. 8). We systematically evaluated coating solutions composed of decanoic acid (C10) and poly-ε-caprolactone (PCL) dissolved in dichloromethane (DCM), with a fixed total solute concentration of 10 wt%. Viscosity measurements using the capillary viscometer method showed that increasing the PCL content significantly raises the solution viscosity due to its polymeric nature (Supplementary Fig. 11b).
During fabrication, a core MNP is dipped into the C10/PCL solution and then withdrawn for solvent evaporation. Higher-viscosity formulations (i.e., low C10:PCL ratios) result in more solution adhering to the microneedle surface, leading to the formation of a thicker shell (Supplementary Fig. 8a). Additionally, partial flow of the viscous solution along the microneedle walls causes a slight decrease in microneedle height and increase in base width (Supplementary Fig. 8c-e). We further tested coating solutions with varying total concentrations at a fixed C10:PCL ratio of 3:1 (Supplementary Fig. 9). As expected, increasing total solute concentration led to a corresponding increase in shell thickness. These results highlight the importance of viscosity control in achieving consistent microneedle geometry across batches.

Supplementary Note 2. Analysis of MNP cargo release kinetics involving reaction-diffusion phenomena

Detailed analysis of the release kinetics from the C10/PCL-mixed formulations revealed two distinct phases with significantly different release rates, governed by the interplay between diffusion-driven mass transport and an effervescent reaction (Supplementary Fig. 10). The first phase (Phase I) is a diffusion-limited regime, where water diffusion through the hydrophobic C10/PCL shell serves as the rate-determining step, resulting in slow cargo release. In the second phase (Phase II), an effervescent reaction at the core-shell interface induces pore formation in the microneedle, significantly accelerating cargo release. The duration of Phase I progressively increased as the C10/PCL ratio decreased (Fig. 2c), indicating that higher PCL content enhances water diffusion resistance, thereby prolonging this phase. Conversely, in formulations with low PCL content, cargo release was dominated by Phase II, where reaction-controlled kinetics dominate due to negligible mass transport resistance for water permeation and cargo hydration. Furthermore, cargo release kinetics could also be tuned by adjusting the fatty acid molecule with different solubility (Supplementary Fig. 17a-c), shell thickness (Supplementary Fig. 17d-f) or HA/$KHCO_3$ ratio of the core (Supplementary Fig. 17g-i). By employing fatty acid with low solubility such as C14, thickening the shell, or increasing the HA/$KHCO_3$ ratio, the cargo release rate could be systematically reduced, demonstrating the programmability of core-shell MNPs to precisely control cargo release kinetics in aqueous environments.

To better understand the cargo release mechanism, we performed additional analysis using the Korsmeyer-Peppas equation (**1**), a widely-used semi-empirical power law equation for describing drug release from polymeric systems:

$$\%\, Cargo\ release = k \times t^n \quad \textbf{(1)}$$

where k is the rate constant, t is the time, and n is the power coefficient.

This model was used to fit cargo release from all microneedle formulations (Supplementary Fig. 16). The results showed that the R^2 values obtained from the Korsmeyer-Peppas fits were consistently lower than those from the first-order kinetic model used in Fig. 2c, indicating that the

first-order model provided a better empirical fit to our data. Among all formulations, the best fit under the Korsmeyer-Peppas model was observed in Supplementary Fig. 16f (C10:PCL = 0:4), with n = 0.50. This result suggests a release mechanism between Fickian and non-Fickian diffusion, and is consistent with our proposed mechanism of cargo release through micropores formed during shell swelling of the microneedle (as illustrated in Supplementary Fig. 11). The better fit of the first-order model compared to the power-law model implies that diffusion is likely the dominant, rate-limiting step under our experimental conditions.

Supplementary Note 3. Computational simulation of cargo release from MNP

Cargo release from the core-shell MNP is driven by a combination of diffusion and effervescent reaction processes. To better understand how these mechanisms influence the spatiotemporal dynamics of cargo release, a mathematical model was constructed. Specifically, a model of two-dimensional core-shell microneedle surrounded by water molecules was developed and the concentration of hydrated cargo (herein denoted as cargo (aq)) was derived using an implicit finite-difference method (Supplementary Fig. 18a). As water from the external environment diffused through the shell, the cargo was gradually hydrated in the core of MNP and then diffused to the outer space through the shell (Supplementary Fig. 18b). This overall cargo release behavior aligns with experimental observations, confirming that diffusive mass transport of water through the shell is necessary to trigger cargo hydration and release from the core.

Finite element analysis was performed for modeling on the cargo-releasing process by Comsol 6.2[1]. Firstly, a simplified model of core-shell MNP was established. The core part included a microneedle (200 µm height and 100 µm base) and a patch base (200 µm width and 50 µm thickness). The thickness of shell was set as 10 µm. A 600 µm x 600 µm of water region was simulated to surround the core-shell MNP model. The components of MNP core and shell were selected according to the actual formulation. The following dissolution processes (**2**-**5**) were simulated:

$$\text{HA (s)} \rightarrow \text{HA (aq)} \quad \textbf{(2)}$$

$$\text{KHCO}_3\text{ (s)} \rightarrow \text{KHCO}_3\text{ (aq)} \quad \textbf{(3)}$$

$$\text{Cargo (s)} \rightarrow \text{Cargo (aq)} \quad \textbf{(4)}$$

$$\text{Cn (s)} \rightarrow \text{Cn (aq)} \quad \textbf{(5)}$$

where “s” and “aq” represented the solid state (before dissolution) and the dissolved state, respectively. “Cargo” is defined to show the typical cargo-releasing behavior from the core-shell MNP. Cn (n = 10, 12 or 14) represented fatty acid. The data of solubility for fatty acids were collected from PubChem (https://pubchem.ncbi.nlm.nih.gov/).

(1) C10: 61.8 mg/L (25 °C)

(2) C12: 4.81 mg/L (25 °C)

(3) C14: 1.07 mg/L (25 °C)

The chemical reaction between $KHCO_3$ and Cn occurred after they were dissolved. The reaction was described as below (**6**). Herein, K-Cn (n = 10, 12 or 14) represented fatty acid potassium salt.

$$KHCO_3\ (aq) + Cn\ (aq) \rightarrow K\text{-}Cn\ (aq) + H_2O + CO_2 \tag{6}$$

The diffusion and reaction process for each component was described as below (**7**).

$$\frac{\partial c}{\partial t} + \nabla \cdot J = R \tag{7}$$

where J and R represented the flux and reacted part of the certain component. J could be further described as below (**8**).

$$J = -D\nabla c \tag{8}$$

where D represented the diffusion coefficient. Typically estimated diffusion coefficients were adopted for components in water (10^{-9} m^2/s) and in the polymer-containing MNP region (2.5×10^{-11} m^2/s)[2,3].

By modelling cargo release across a cross-sectional area representing water-shell-core interfaces, computational simulation revealed that water infiltration into MNP shell was slow in the first 3 minutes due to the shell hydrophobicity (Supplementary Fig. 18c). During this time, cargo hydration and the reaction between C10 and $KHCO_3$ were suppressed. This simulated delay time is consistent with experimental observations of the RhB cargo-release kinetics (Fig. 2c) Water diffusion through the shell accelerated in the following 30 seconds, leading to hydration of the cargo inside the core (Supplementary Fig. 18c). Such phenomenon resulted from the reaction between C10 and $KHCO_3$ at the core-shell boundary, producing effervescent CO_2 bubbles and K-C10 salt and facilitating cargo hydration. This was corroborated by the elevated cargo (aq) concentration in the simulation model. The effervescent reaction triggered a burst release of the cargo as shown by the negligible cargo (aq) concentration after the reaction (Supplementary Movie 1). The simulated cargo-release kinetics showed good agreement with the experimental results (Supplementary Fig. 19d). This model could also be used to predict cargo release kinetics and guide microneedle design parameters, such as the core or shell formulations. Increasing the PCL content in the shell would lead to a prolonged duration of Phase I and a lower release rate in Phase

II (Supplementary Fig. 18e, f). A similar effect could be realized by increasing the shell thickness (Supplementary Fig. 18g, h), employing low-solubility fatty acids (Supplementary Fig. 18i, j) or reducing the $KHCO_3$ content in the core (Supplementary Fig. 18k, l).

Supplementary Note 4. Water contact angle measurement and FTIR analysis

The water-responsive cargo release mechanism was further investigated through water contact angle measurements. Fatty acids, characterized by shorter carbon chains and hydrophilic carboxyl groups, are typically less hydrophobic than PCL. As expected, increasing PCL content in the shell led to higher water contact angles, ranging from 64.2° to 120.0° (Supplementary Fig. 19a), This indicates increased resistance to water infiltration through the MNP shell, consistent with the prolonged Phase I duration observed at higher PCL concentrations. Fatty acid with a longer carbon chain demonstrated greater hydrophobicity (Supplementary Fig. 19b). In contrast, the mixture of HA and $KHCO_3$ in the core components of MNP exhibited pronounced hydrophilicity (Supplementary Fig. 19c). To better mimic the core-shell MNP design, contact angle measurements on two-layer samples were also investigated. Interestingly, the water contact angle stabilized around 110° when using pure PCL as the hydrophobic layer (Fig. 2e). The water contact angle gradually decreased as more C10 was incorporated. Such phenomenon was also observed in other formulations containing both fatty acid and $KHCO_3$, which could be attributed to the formation of fatty acid-potassium salt acting as a surfactant (Supplementary Fig. 19d, e). FTIR spectra revealed a typical asymmetric stretching vibration peak of carboxylate at 1549 cm^{-1}, consistent with the formation of potassium decanoate (K-C10) after the effervescent reaction (Fig. 2f, Supplementary Fig. 20).

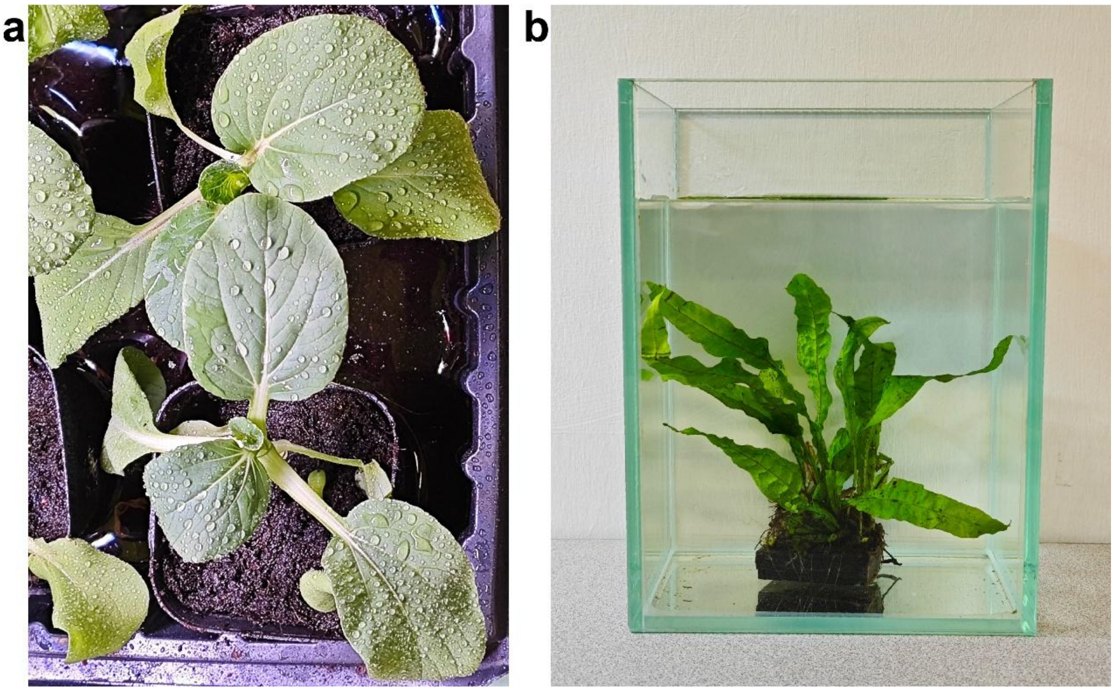

Supplementary Fig. 1. Representative wet environments that plants may encounter. (a) Photograph of wet leaf surfaces of a typical terrestrial plant after watering. (b) Photograph of an aquatic plant immersed underwater.

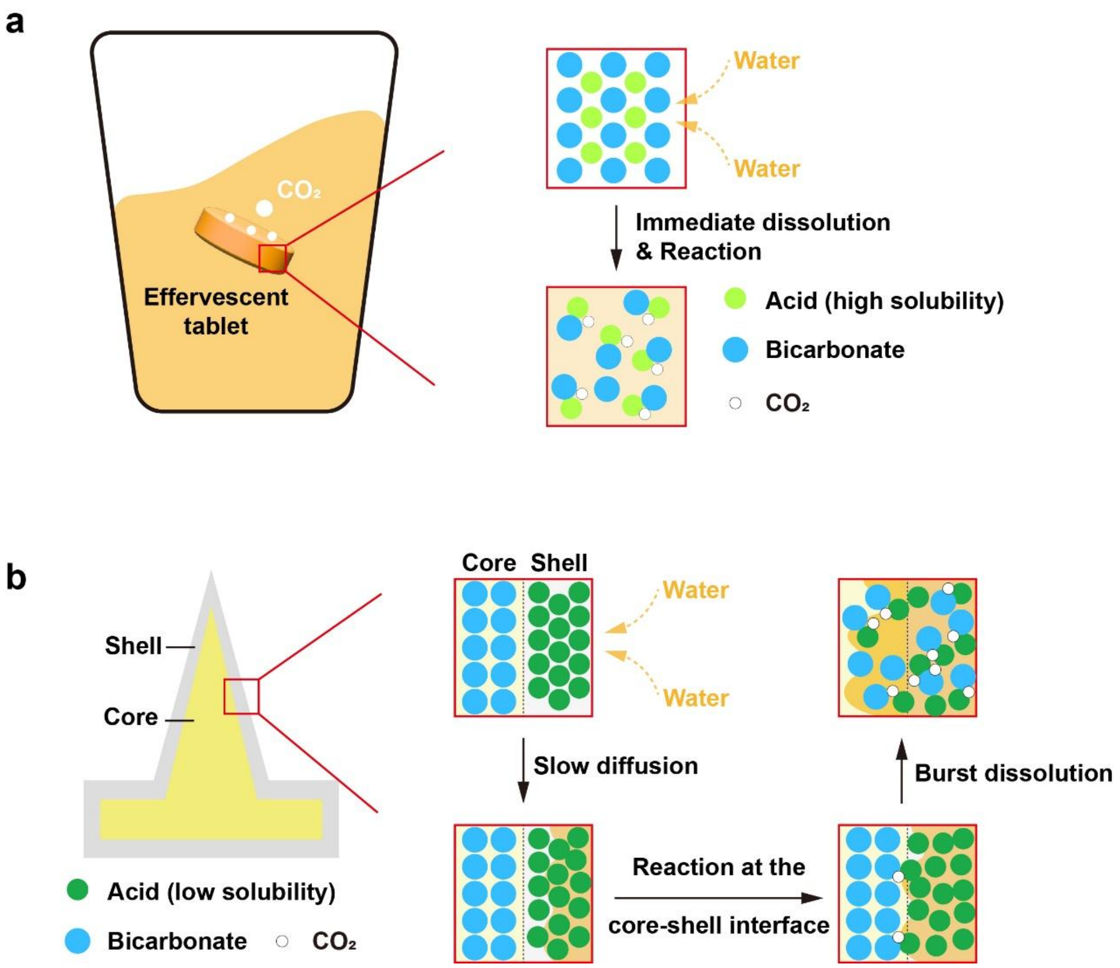

Supplementary Fig. 2. Schematic illustration comparing the distinct effervescent mechanisms. (a) Typical effervescent medicinal tablet. (b) Water-responsive MNP developed in this work.

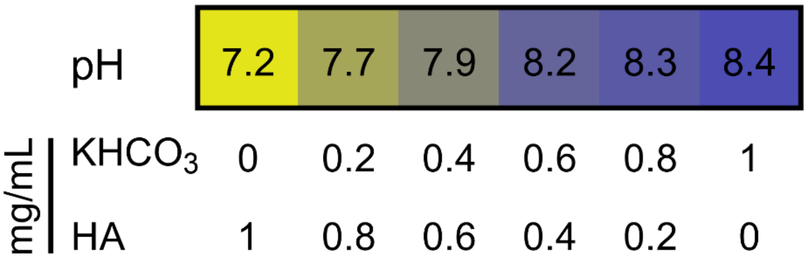

Supplementary Fig. 3. pH values of HA, $KHCO_3$ and their mixed solution. The total concentration of HA and $KHCO_3$ was set to 1 mg/mL for each solution.

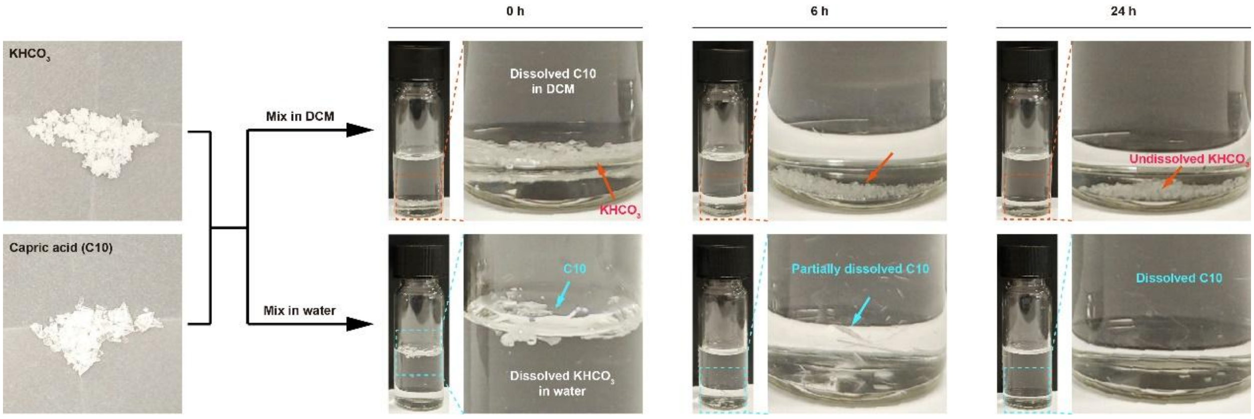

Supplementary Fig. 4. Photographs of $KHCO_3$ and C10 solid powders upon dissolution in DCM or water. In DCM, only C10 dissolved. In water, $KHCO_3$ quickly dissolved and C10 slowly dissolved in 24 hours.

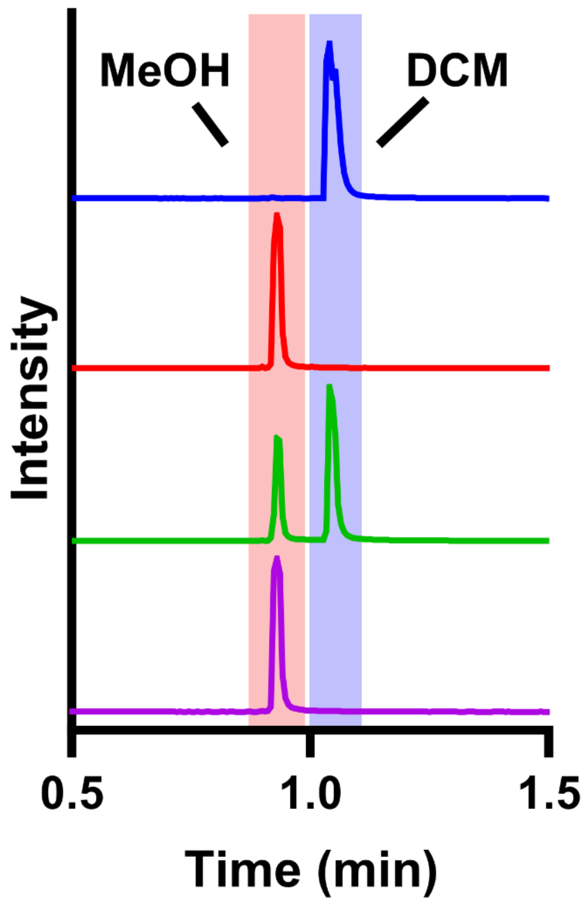

Supplementary Fig. 5. GC/MS spectra of MNP preparation in different solvent. Spectra for DCM, methanol (MeOH), mixture of DCM and MeOH and the extraction from the core-shell MNP (extracted by MeOH), from top to bottom. Source data are provided as a Source Data file.

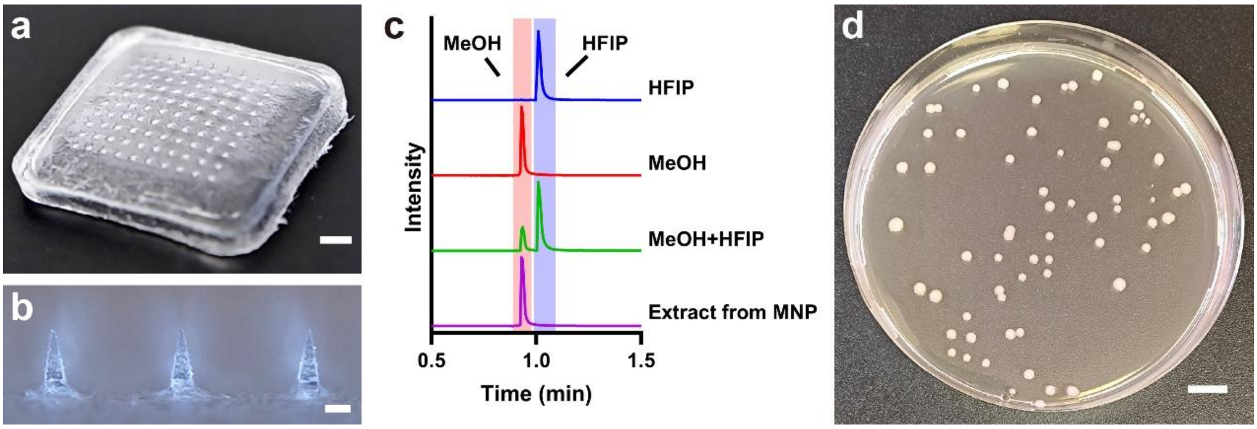

Supplementary Fig. 6. HFIP as an alternative solvent in core-shell MNP preparation. (a)-(b) Images of the core-shell MNP (scale bar, 1 mm, a; 100 μm, b). (c) GC/MS spectra for HFIP, MeOH, mixture of HFIP and MeOH and the extraction from the core-shell MNP (extracted by MeOH). (d) Bacterial colonies of *A. tumefaciens* from *A. tumefaciens*-loaded core-shell MNP (scale bar, 1 cm). Source data are provided as a Source Data file.

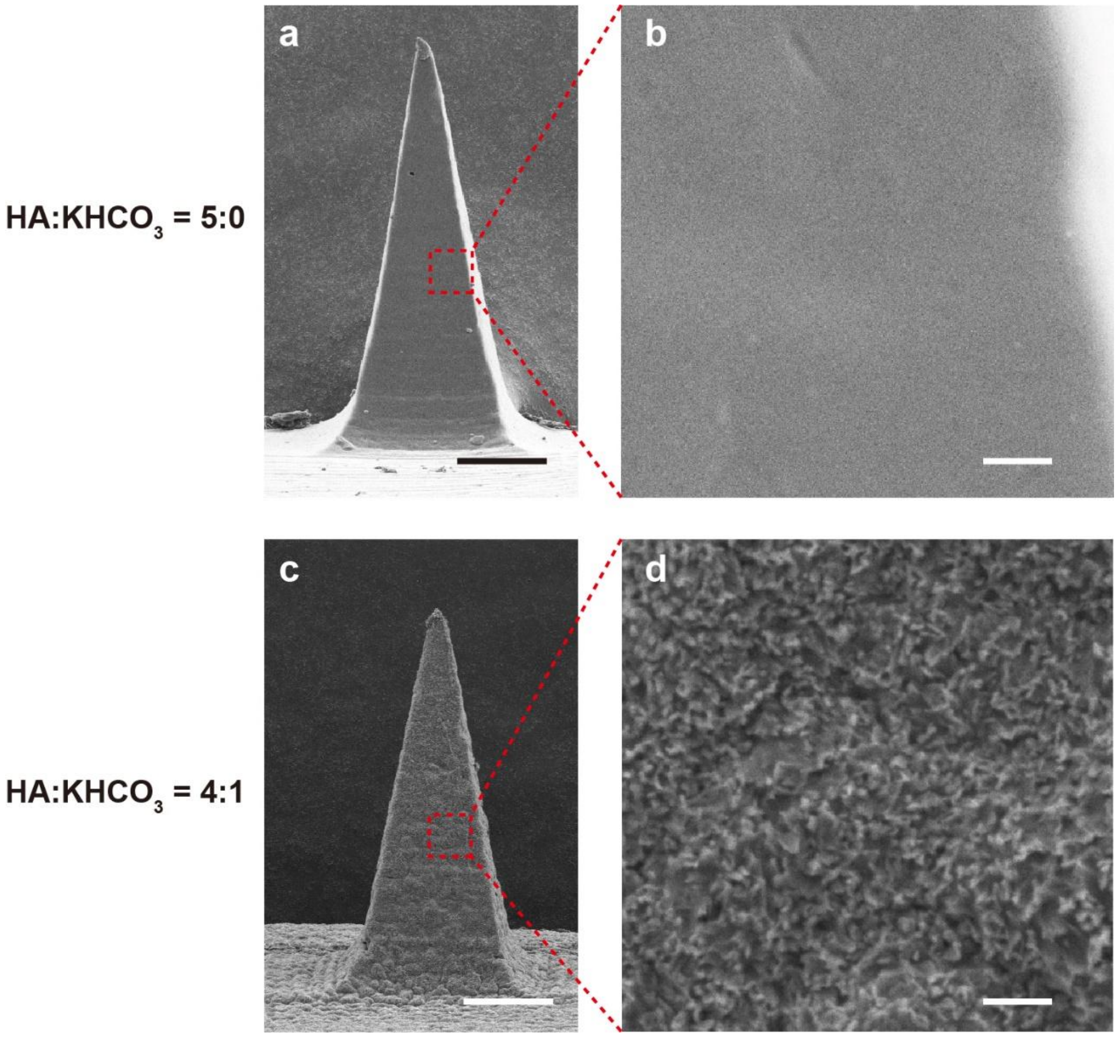

Supplementary Fig. 7. SEM images of MNPs. MNPs were prepared by (a, b) HA and (c, d) HA/$KHCO_3$ mixture (HA:$KHCO_3$ = 4:1, w/w). Scale bars, 50 µm for (a) and (c); 2 µm for (b) and (d).

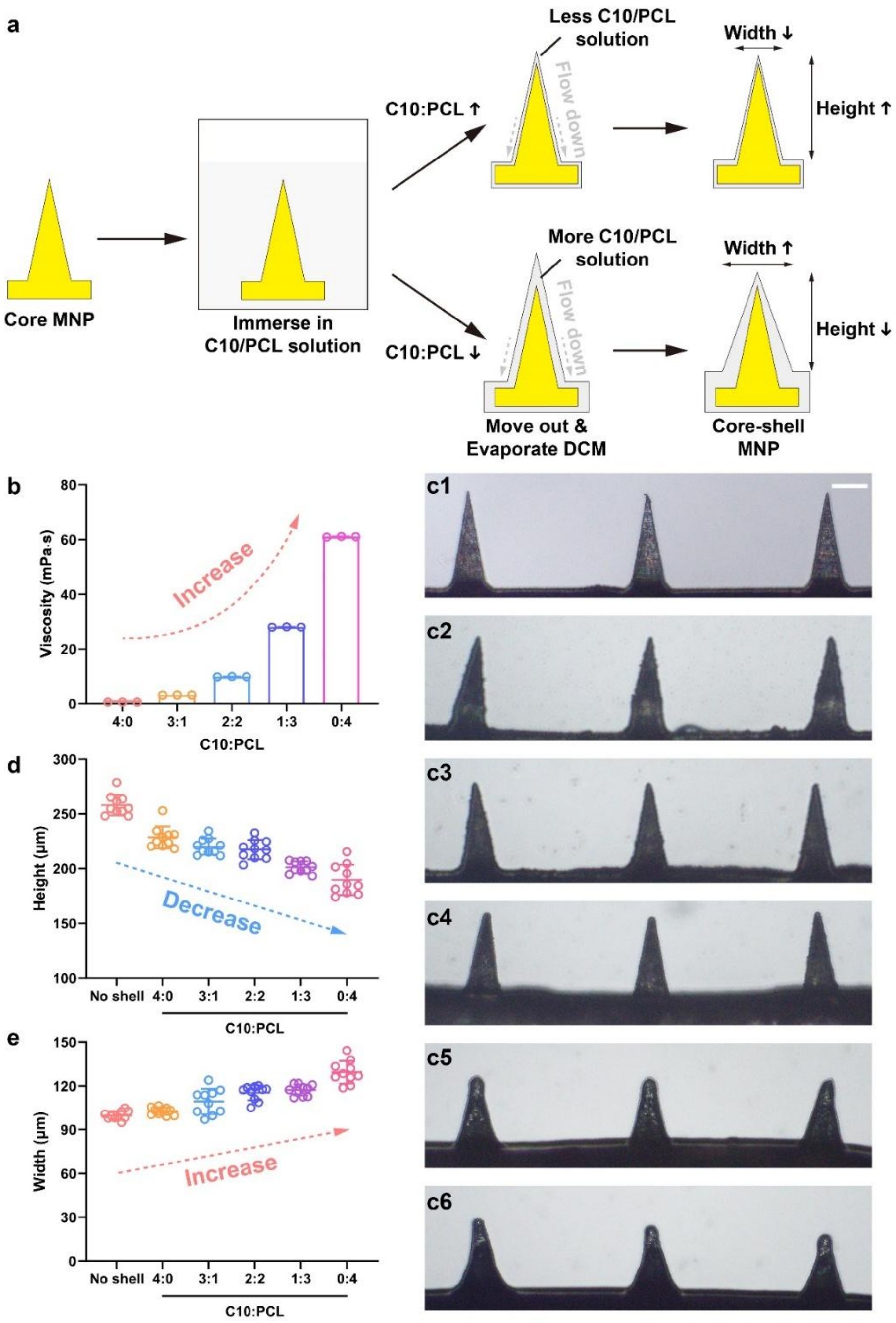

Supplementary Fig. 8. Preparation and morphology analysis of core-shell MNPs. (a) Schematic illustration of the formation of shell of the core-shell MNP. (b) Viscosity values of C10/PCL solutions under different ratios. (c) Images of the core MNP (c1) and core-shell MNPs with ratios of C10:PCL = 4:0 (c2), 3:1 (c3), 2:2 (c4), 1:3 (c5) and 0:4 (c6) (scale bar, 100 μm). (d) Heights and (e) widths of microneedles from the core MNP ("no shell" group) and core-shell MNPs. All the total concentrations of C10 and PCL used in (b)-(e) was 10wt% in DCM. Data in (b), (d) and (e) were shown as mean ± s.d. (n = 3 independent samples, b; n = 10 independent samples, d, e). Source data are provided as a Source Data file.

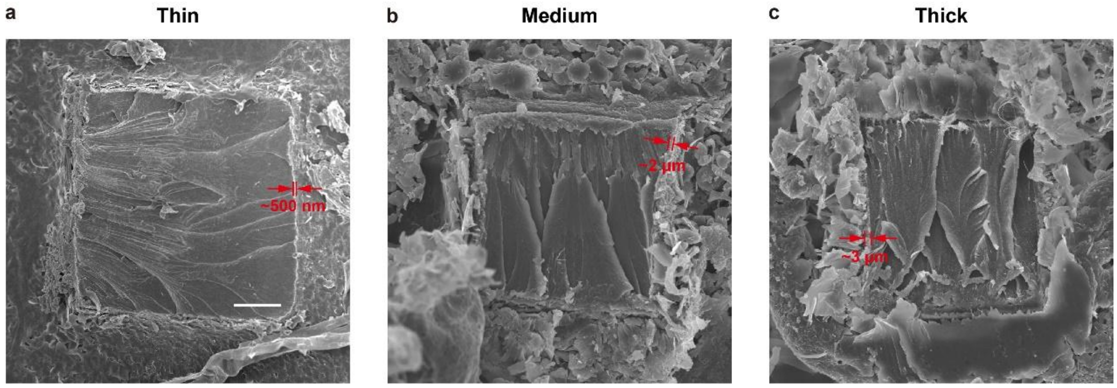

Supplementary Fig. 9. SEM images for the core-shell microneedles with the thickness of thin, medium and thick shell. The core consisted of HA and $KHCO_3$ (HA:$KHCO_3$ = 4:1, w/w). The shell consisted of C10 and PCL (C10:PCL = 3:1, w/w). The total concentration of C10/PCL mixture in DCM, in terms of weight percentage, was (a) 5%, (b) 10%, and (c) 20% when preparing the core-shell MNPs. Scale bar, 20 µm.

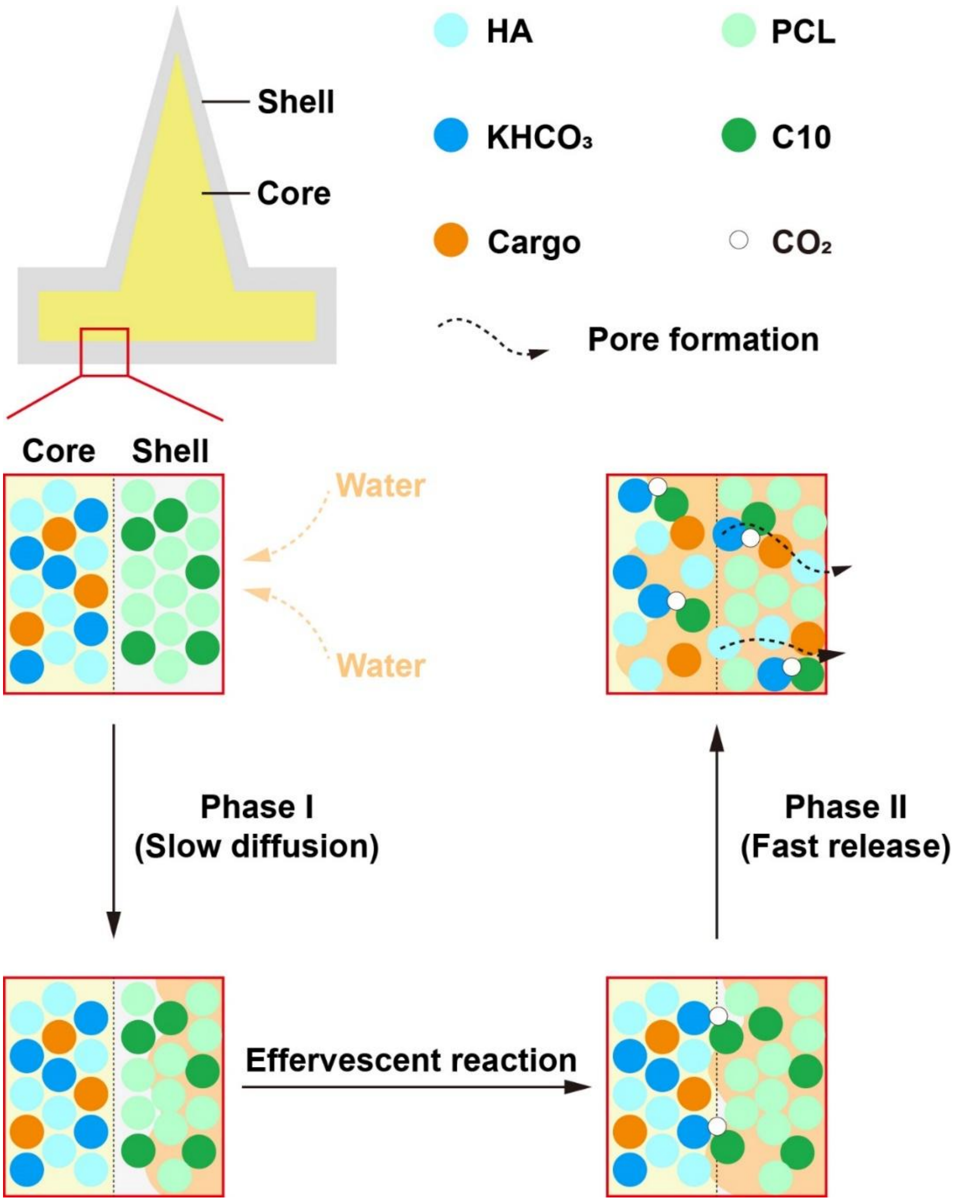

Supplementary Fig. 10. Schematic illustration of two-phase-model cargo release from the core-shell MNP.

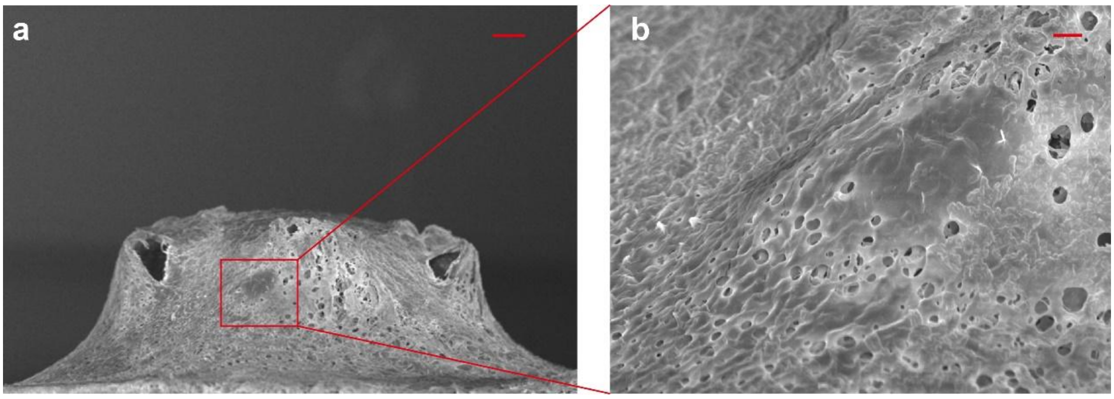

Supplementary Fig. 11. SEM images for the water-treated core-shell microneedle. (a) low magnification (scale bar, 10 μm) and (b) high magnification (scale bar, 2 μm). The core composed of HA and $KHCO_3$ (HA:$KHCO_3$ = 4:1, w/w). The shell composed of C10 and PCL (C10:PCL = 1:3, w/w). The microneedle was immersed in water for 5 minutes and then freeze-dried.

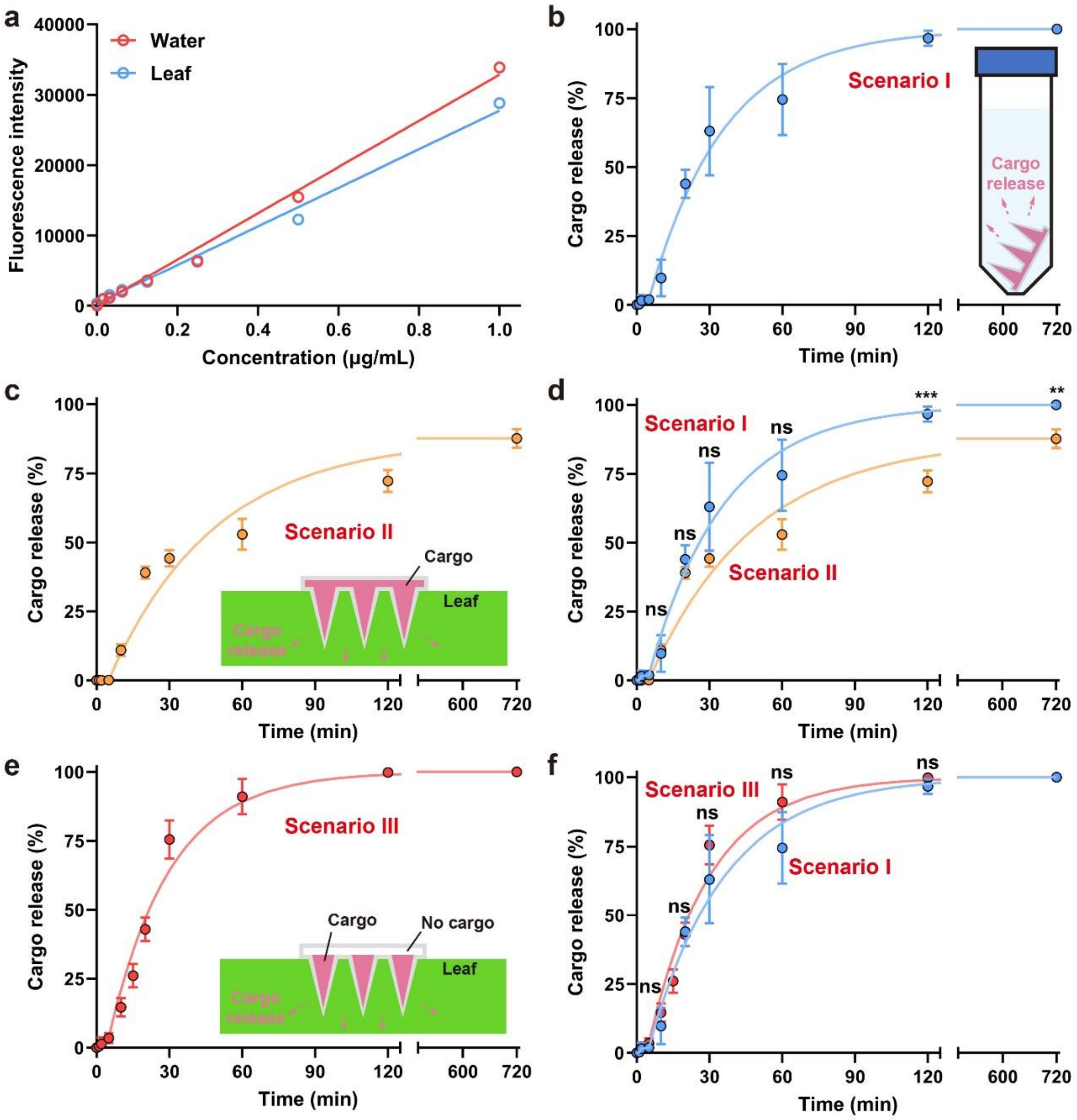

Supplementary Fig. 12. Release profiles of the core-shell MNP in water and leaves. (a) Calibration curve of RhB in water or in the supernatant obtained from homogenized leaf tissue. *In vitro* release profiles of the core-shell MNP (C10:PCL = 1:3) under conditions of (b) full immersion in water (Scenario I, same as the "C10:PCL = 1:3" group in **Fig. 2c**) and (c) insertion into leaf tissue (Scenario II). (d) Comparison between Scenario I and II. (e) *In vitro* release profiles of the core-shell MNP (C10:PCL = 1:3, with cargo loaded only in the microneedle tips) under the condition of insertion into leaf tissue (Scenario III). (f) Comparison between Scenario I and III. Data in (b)-(f) were shown as mean ± s.d. (n = 3 independent samples). Statistical analysis was performed by two-tailed Student's t-test. ns, $P \geq 0.05$, $^{**}P < 0.01$ and $^{***}P < 0.001$. Source data are provided as a Source Data file.

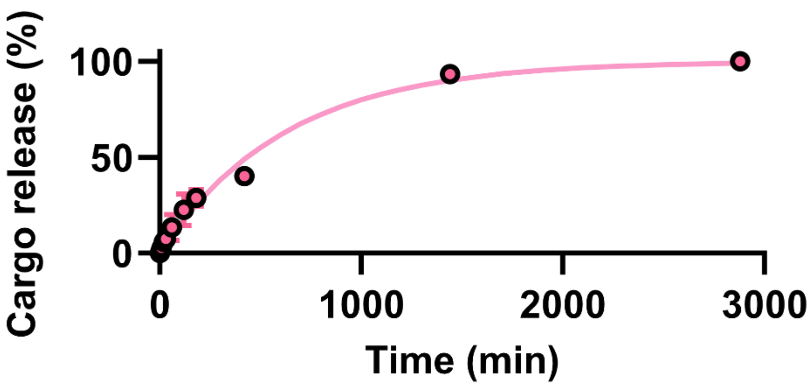

Supplementary Fig. 13. ***In vitro*** **cargo release profile of the core-shell MNP with PCL shell over 2 days.** Data were shown as mean ± s.d. (n = 3 independent samples). Source data are provided as a Source Data file.

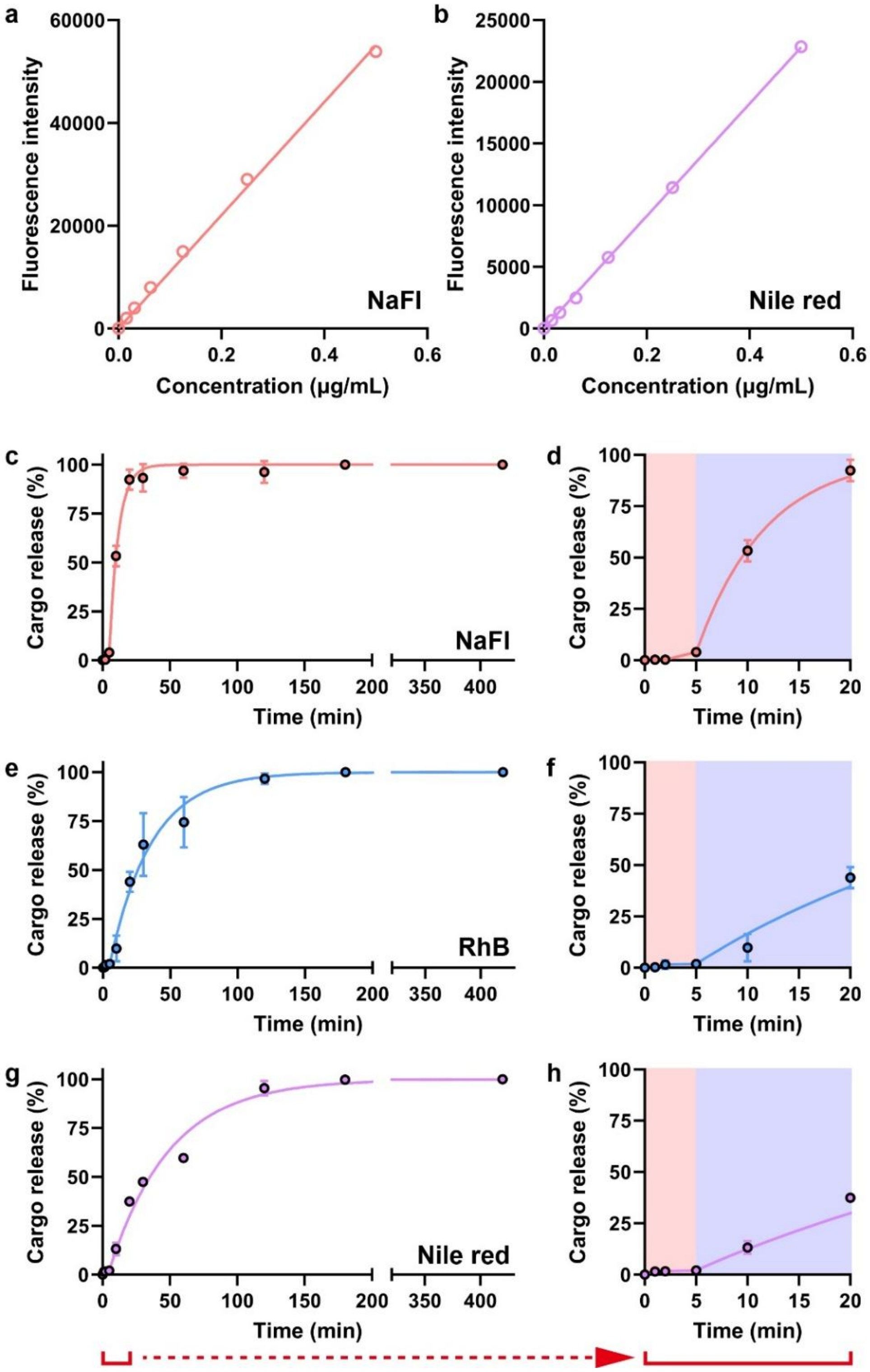

Supplementary Fig. 14. ***In vitro*** **release profiles of core-shell MNPs loaded with different cargoes.** Calibration curve of (a) fluorescein sodium salt (NaFl) and (b) Nile red. *In vitro* release profiles of the core-shell MNP (C10:PCL = 1:3) loaded with cargo of (c)-(d) NaFl, (e)-(f) RhB and (g)-(h) Nile red. Data in (c)-(h) were shown as mean ± s.d. (n = 3 independent samples). Source data are provided as a Source Data file.

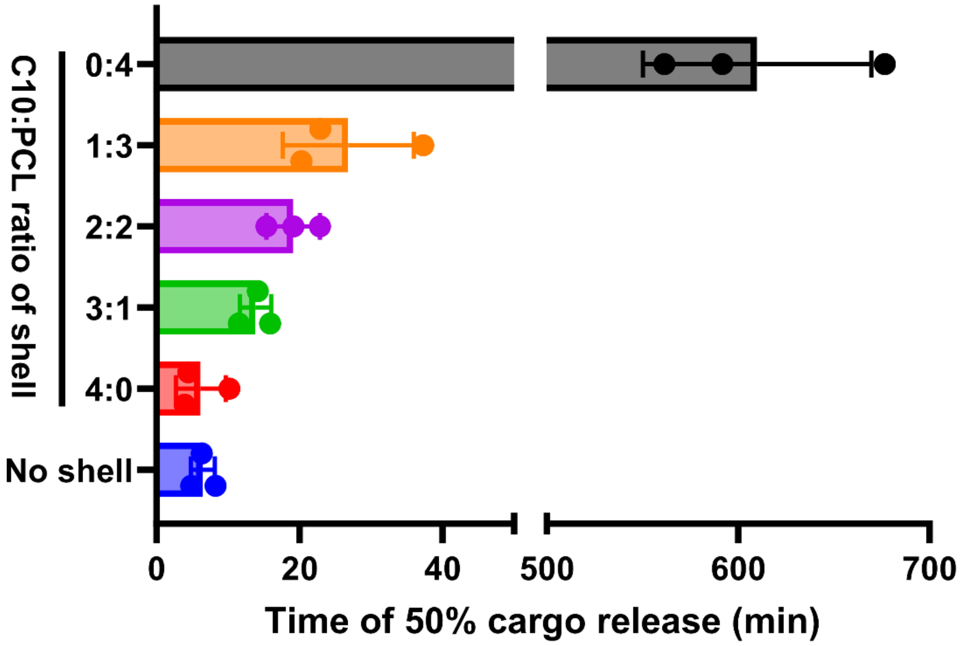

Supplementary Fig. 15. Time taken for 50% of the loaded cargo to be released for the core-MNP and different core-shell MNPs. Data were shown as mean ± s.d. (n = 3 independent samples). Source data are provided as a Source Data file.

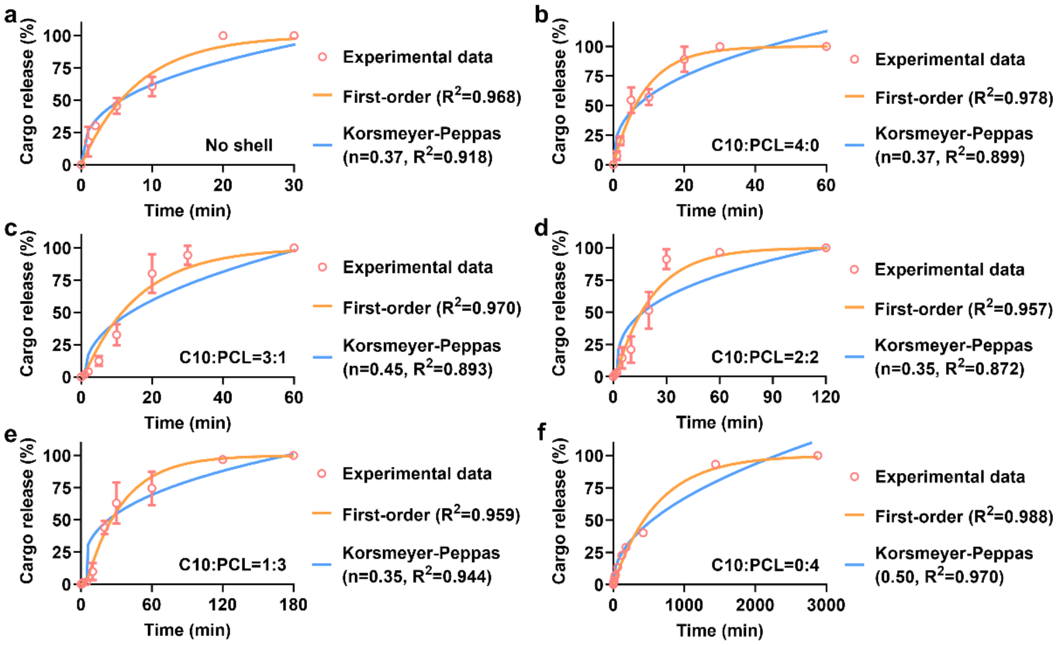

Supplementary Fig. 16. Comparison of first-order kinetic model and Korsmeyer-Peppas model on the in-vitro-releasing experimental data. Experimental data was collected from core MNP (a) and core-shell MNPs. with ratios of C10:PCL = 4:0 (b), 3:1 (c), 2:2 (d), 1:3 (e) and 0:4 (f). Data of (c), (e) and (h) represent mean ± s.d. (n = 3 independent samples). Source data are provided as a Source Data file.

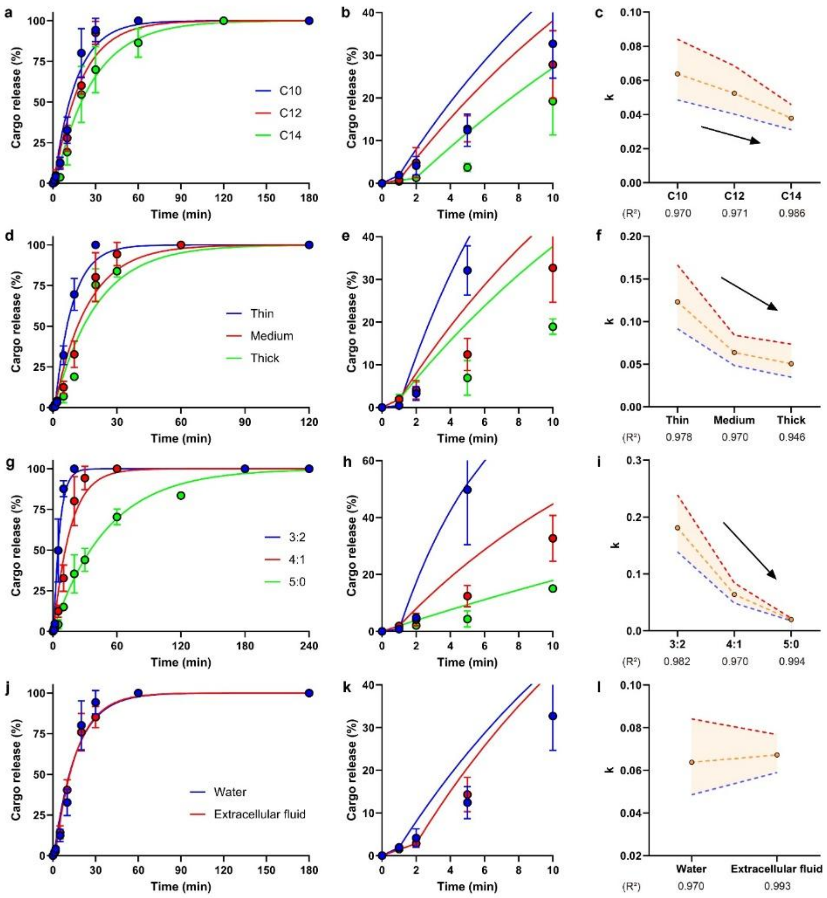

Supplementary Fig. 17. The *in vitro* release profiles of the core-shell MNPs and fitted k (rate constants). k values were fitted according to the first-order release kinetics. Data of (a), (b), (d), (e), (g), (h), (j) and (k) were shown as mean ± s.d. (n = 3 independent samples). In (c), (f), (i) and (l), the upper and lower lines represented the upper and lower limits of the 95% confidence interval; the data points in the middle represented the best fitted values of k. (a)-(c) Core: HA:$KHCO_3$ = 4:1 (w/w); shell: fatty acid:PCL = 3:1 (w/w). The total concentration of fatty acid/PCL mixture in DCM was 10wt% when preparing the core-shell MNPs. Fatty acids included C10, C12 or C14. Media: water. (d)-(f) Core: HA:$KHCO_3$ = 4:1 (w/w); shell: C10:PCL = 3:1 (w/w). The total concentration of C10/PCL mixture in DCM was 5wt% (thin), 10wt% (medium) and 20wt% (thick) when preparing the core-shell MNPs. Media: water. (g)-(i) Core: HA:$KHCO_3$ = 3:2, 4:1 or 5:0 (w/w); shell: C10:PCL = 3:1 (w/w). The total concentration of C10/PCL mixture in DCM was 10wt% when preparing the core-shell MNPs. Media: water. (j)-(l) Core: HA:$KHCO_3$ = 4:1 (w/w); shell: C10:PCL = 3:1 (w/w). The total concentration of C10/PCL mixture in DCM was 10wt% when preparing the core-shell MNPs. Media: water or extracellular fluid. Source data are provided as a Source Data file.

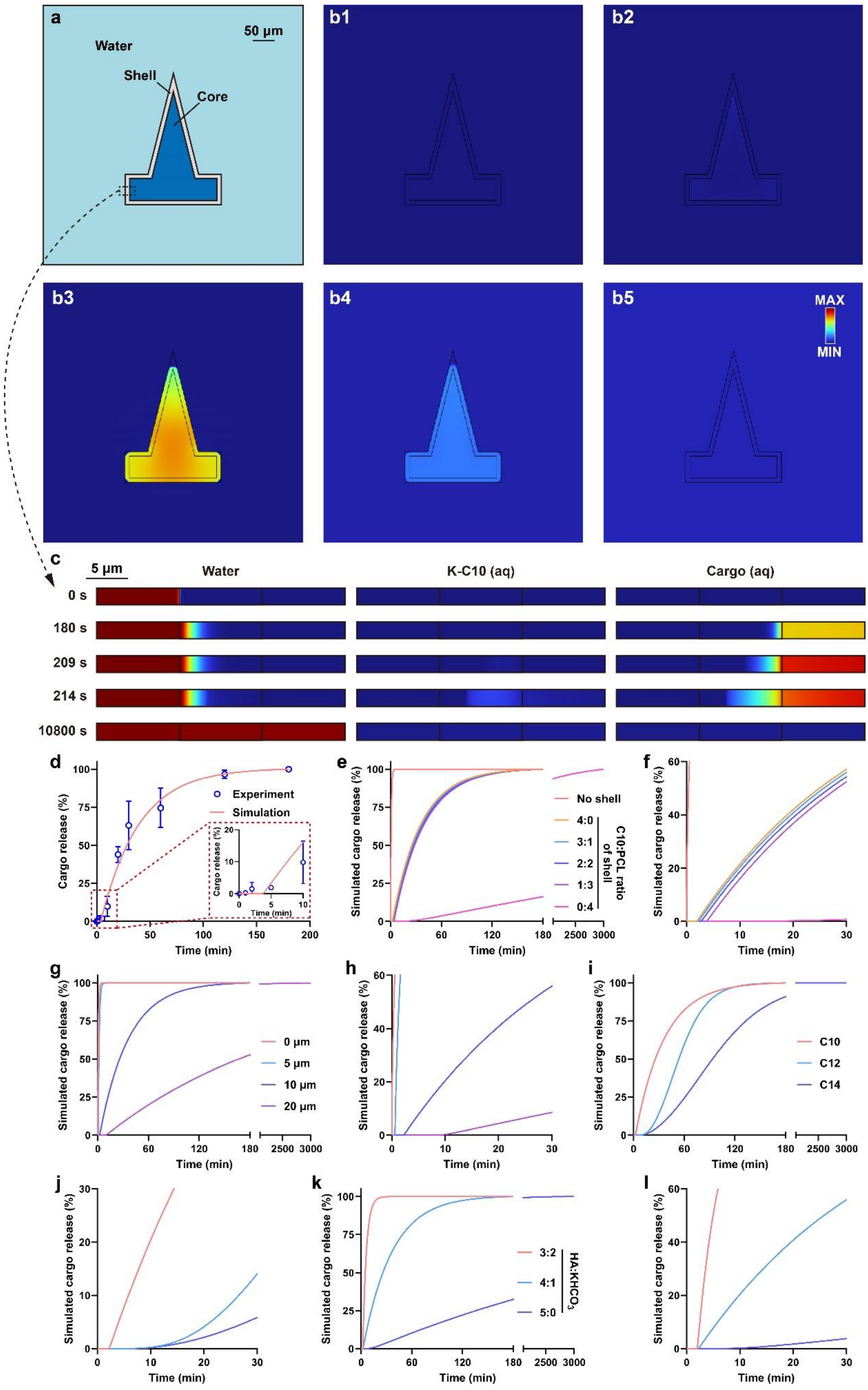

Supplementary Fig. 18. Computational modeling of cargo release from the core-shell

microneedle. (a) Two-dimensional model of a core-shell microneedle and the surrounding water. The content mapping of cargo (aq) with the time sequence of (b1) 0 minute, (b2) 2 minutes, (b3) 5 minutes, (b4) 30 minutes and (b5) 3 hours. (c) Time-sequence mapping for the contents of water, K-C10 (aq) and cargo (aq) in the 2 μm x 30 μm of area. The area was evenly divided into 3 sections (2 μm x 10 μm for each) from left to right, representing water, shell and core regions, sequentially. The core components involved HA, $KHCO_3$ and cargo (4:1:0.01, w/w), and shell components involved C10 and PCL (1:3, w/w). (d) Experimental data (means ± s.d., n = 3 independent samples) and the simulating releasing profile of the core-shell MNP. Core: HA:$KHCO_3$ = 4:1 (w/w); shell: C10:PCL = 1:3 (w/w). (e)-(f) Simulated cargo-releasing profiles of core MNP ("no shell") and core-shell MNPs with different ratios of C10:PCL. Core: HA:$KHCO_3$ = 4:1 (w/w). (g)-(h) Simulated cargo-releasing profiles of core-shell MNPs with different shell thickness. Core: HA:$KHCO_3$ = 4:1 (w/w); shell: C10:PCL = 3:1 (w/w). (i)-(j) Simulated cargo-releasing profiles of core-shell MNPs with different fatty acid. Core: HA:$KHCO_3$ = 4:1 (w/w); shell: fatty acid:PCL = 3:1 (w/w). (k)-(l) Simulated cargo-releasing profiles of core-shell MNPs with different ratios of HA:$KHCO_3$. Shell:C10:PCL = 3:1 (w/w). Source data are provided as a Source Data file.

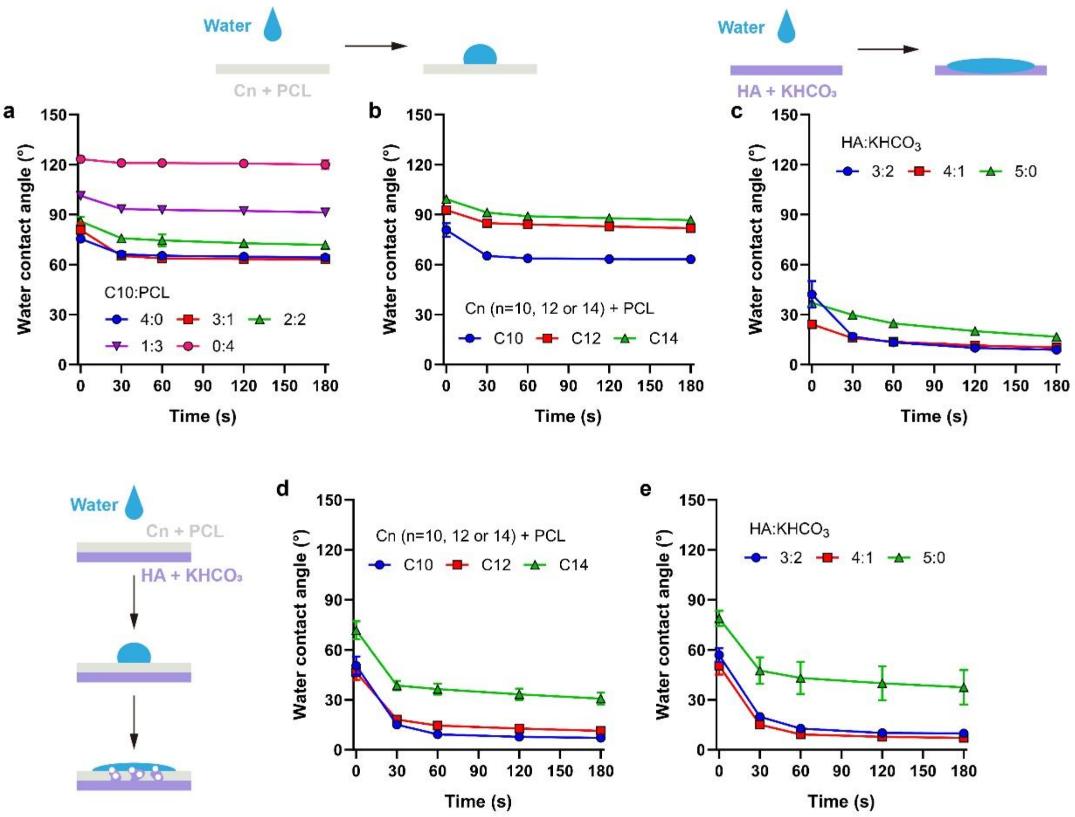

Supplementary Fig. 19. Water contact angle measurements for different MNP formulations. (a) Time-profile of water contact angle on a hydrophobic layer containing C10 and PCL with ratios ranging from 4:0 to 0:4 (w/w). (b) Time-profile of water contact angle on a hydrophobic layer containing different fatty acids and PCL with constant fatty acid:PCL (3:1, w/w). (c) Time-profile of water contact angle on a hydrophilic layer containing HA and $KHCO_3$ with ratios ranging from 5:0 to 3:2 (w/w). (d) Time-profile of water contact angle on double layer samples to mimic microneedle design. The upper layer contained fatty acid:PCL (3:1, w/w). The lower layer contained HA:$KHCO_3$ 4:1 (w/w). (e) Time-profile of water contact angle on double layer samples, with the upper layer containing C10:PCL (3:1, w/w). The lower layer contained HA and $KHCO_3$ with different ratios. Data were shown as mean ± s.d. (n = 3 independent samples). Source data are provided as a Source Data file.

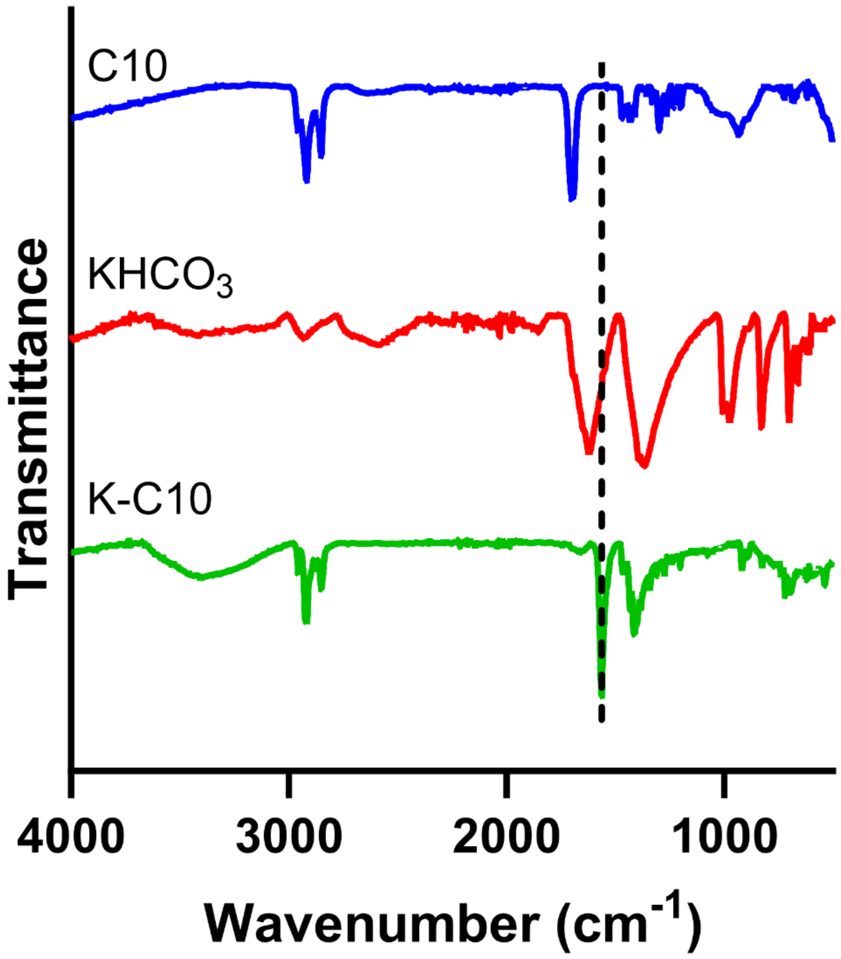

Supplementary Fig. 20. FTIR spectra of C10, $KHCO_3$ and K-C10. Source data are provided as a Source Data file.

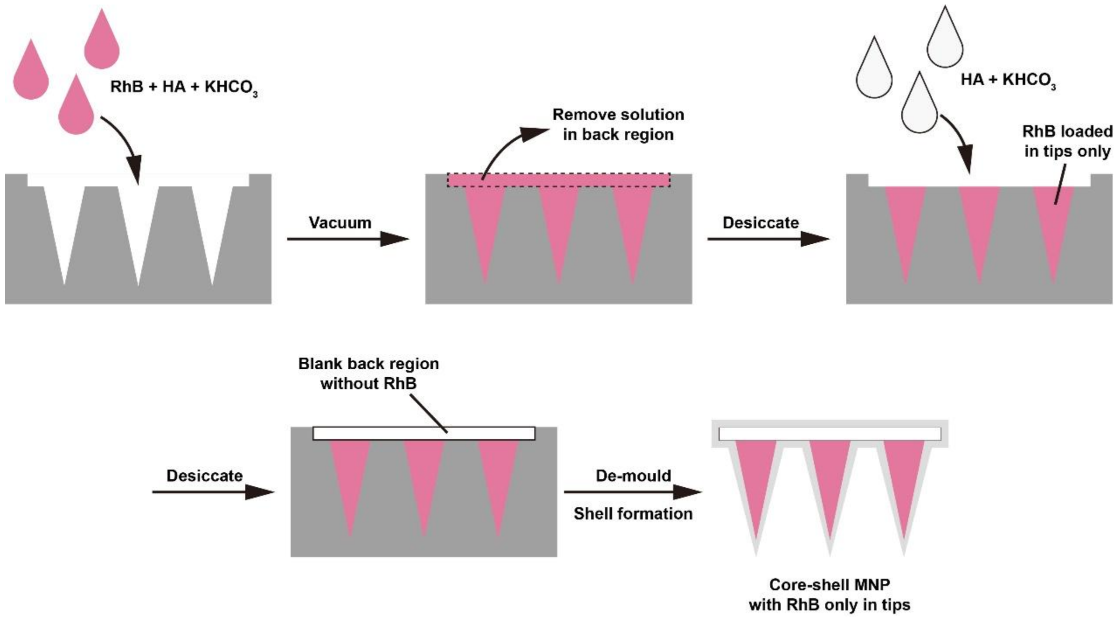

Supplementary Fig. 21. Schematic illustration of the preparation process of the core-shell MNP with cargo (RhB) only in microneedle tips.

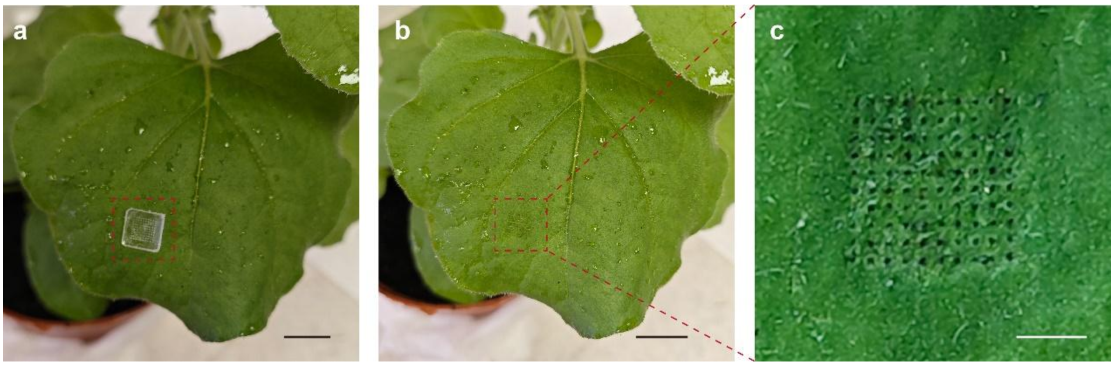

Supplementary Fig. 22. Application of core-shell MNP on the wet leaf of *N. benthamiana*. (a) A core-shell MNP was applied on the wet leaf of *N. benthamiana*. (b) Micropores were formed by MNP on the leaf surface. (c) Leaf micropores were stained by trypan blue for visualization. Scale bar, 1 cm (a, b); 2 mm (c).

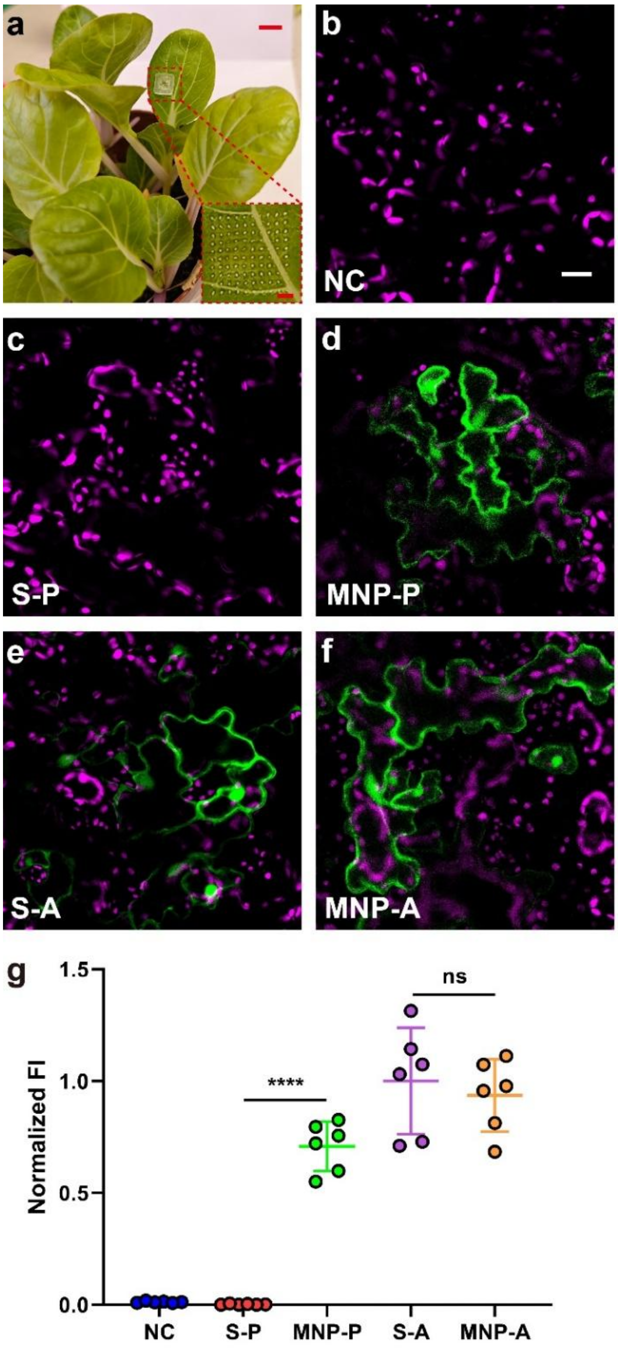

Supplementary Fig. 23. Delivery of GFP-encoding plasmid DNA and active *A. tumefaciens* on *B. chinensis*. (a) Images showing the application of core-shell MNP on the *B. chinensis* leaf (scale bar, 1 cm, out of the dashed box; 1 mm, in the dashed box). Representative confocal images of *B. chinensis* from groups of (b) NC, (c) S-P, (d) MNP-P, (e) S-A and (f) MNP-A (scale bar, 10 μm). (g) Quantification of GFP fluorescence intensity of different treatment groups on *B. chinensis*. Data represent mean ± s.d. (n = 6 independent biological replicates). Statistical analysis was performed by one-way ANOVA with Tukey's multiple comparisons test. ns $P \geq 0.05$ and ****$P < 0.0001$. Source data are provided as a Source Data file.

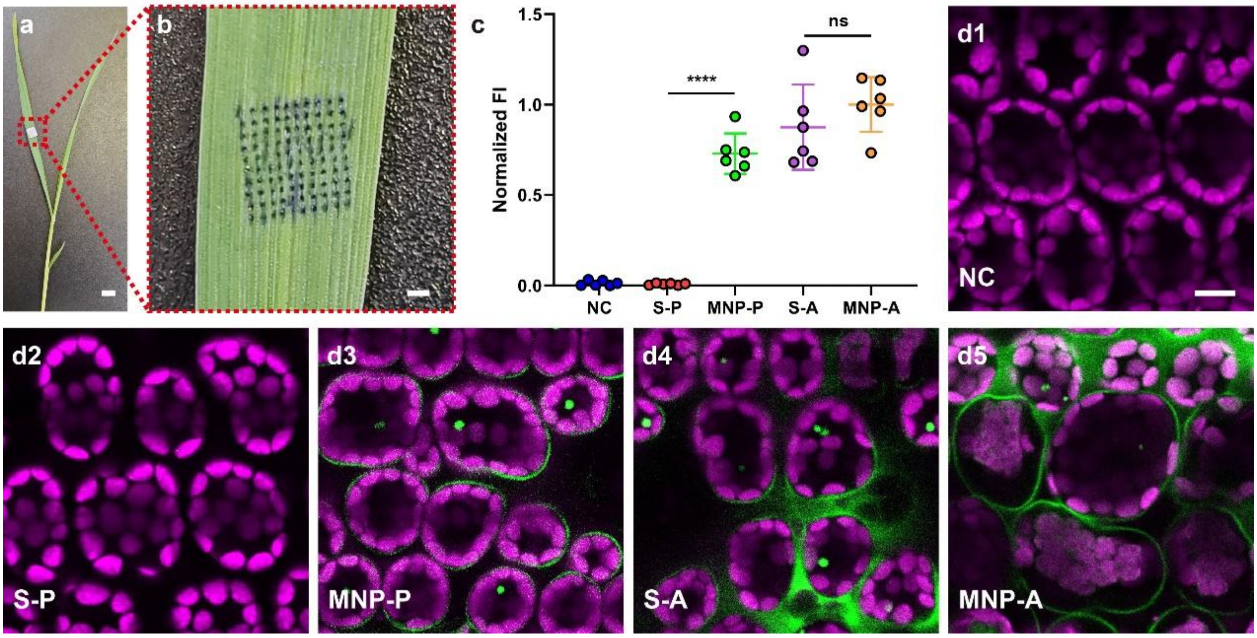

Supplementary Fig. 24. Delivery of GFP-encoding plasmid DNA and active *A. tumefaciens* on *H. vulgare*. (a) Images showing the application of core-shell MNP on the *H. vulgare* leaf (scale bar, 1 cm). (b) Trypan blue staining image of micropores formed on the leaf surface of *H. vulgare* after MNP application (scale bar, 1 mm). (c) Quantification of GFP fluorescence intensity of different treatment groups on *H. vulgare*. Representative confocal images of *H. vulgare* leaves from groups of (d1) NC, (d2) S-P, (d3) MNP-P, (d4) S-A and (d5) MNP-A (scale bar, 10 μm). Data represent mean ± s.d. (n = 6 independent biological replicates). Statistical analysis was performed by one-way ANOVA with Tukey's multiple comparisons test. ns $P \geq 0.05$ and ****$P < 0.0001$. Source data are provided as a Source Data file.

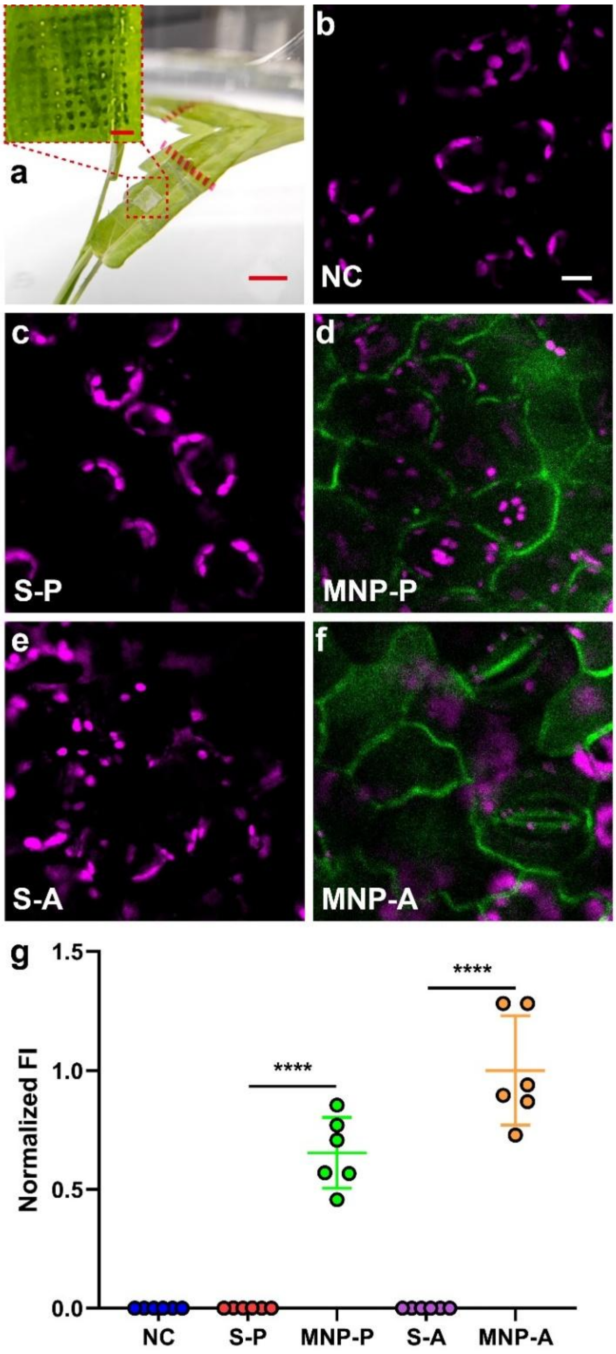

Supplementary Fig. 25. Delivery of GFP-encoding plasmid DNA and active *A. tumefaciens* on *I. aquatica*. (a) Images showing the application of core-shell MNP on the *I. aquatica* leaf under the submerged condition (scale bar, 1 cm, out of the dashed box; 1 mm, in the dashed box). Representative confocal images of *I. aquatica* from groups of (b) NC, (c) S-P, (d) MNP-P, (e) S-A and (f) MNP-A (scale bar, 10 μm). (g) Quantification of GFP fluorescence intensity of different treatment groups on *I. aquatica*. Data represent mean ± s.d. (n = 6 independent biological replicates). Statistical analysis was performed by one-way ANOVA with Tukey's multiple comparisons test. ****$P < 0.0001$. Source data are provided as a Source Data file.

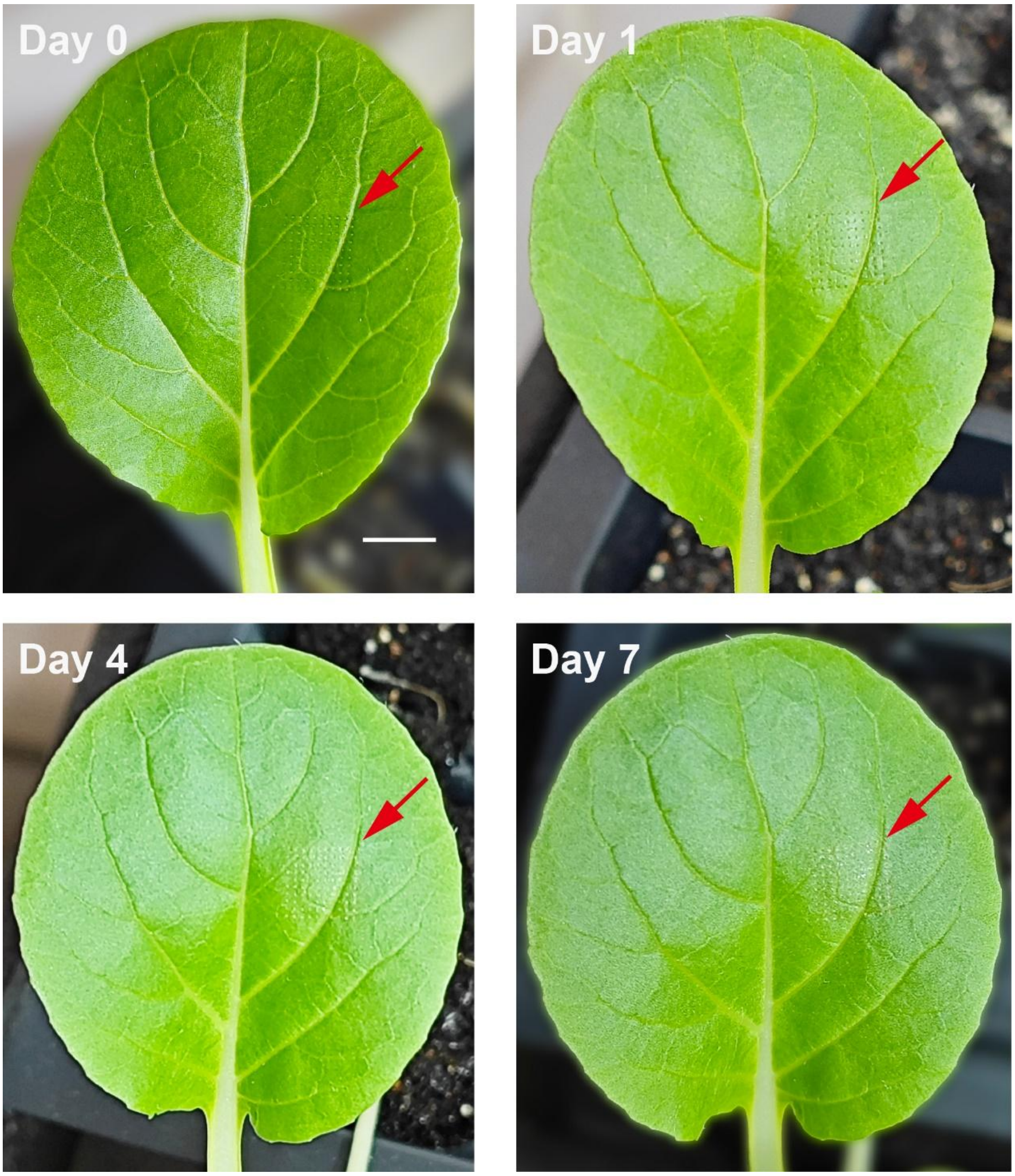

Supplementary Fig. 26. Time-sequence images of a *B. chinensis* leaf over 7 days after treatment with core-shell MNP. The treated area was marked by the red arrow. Scale bar, 5 mm.

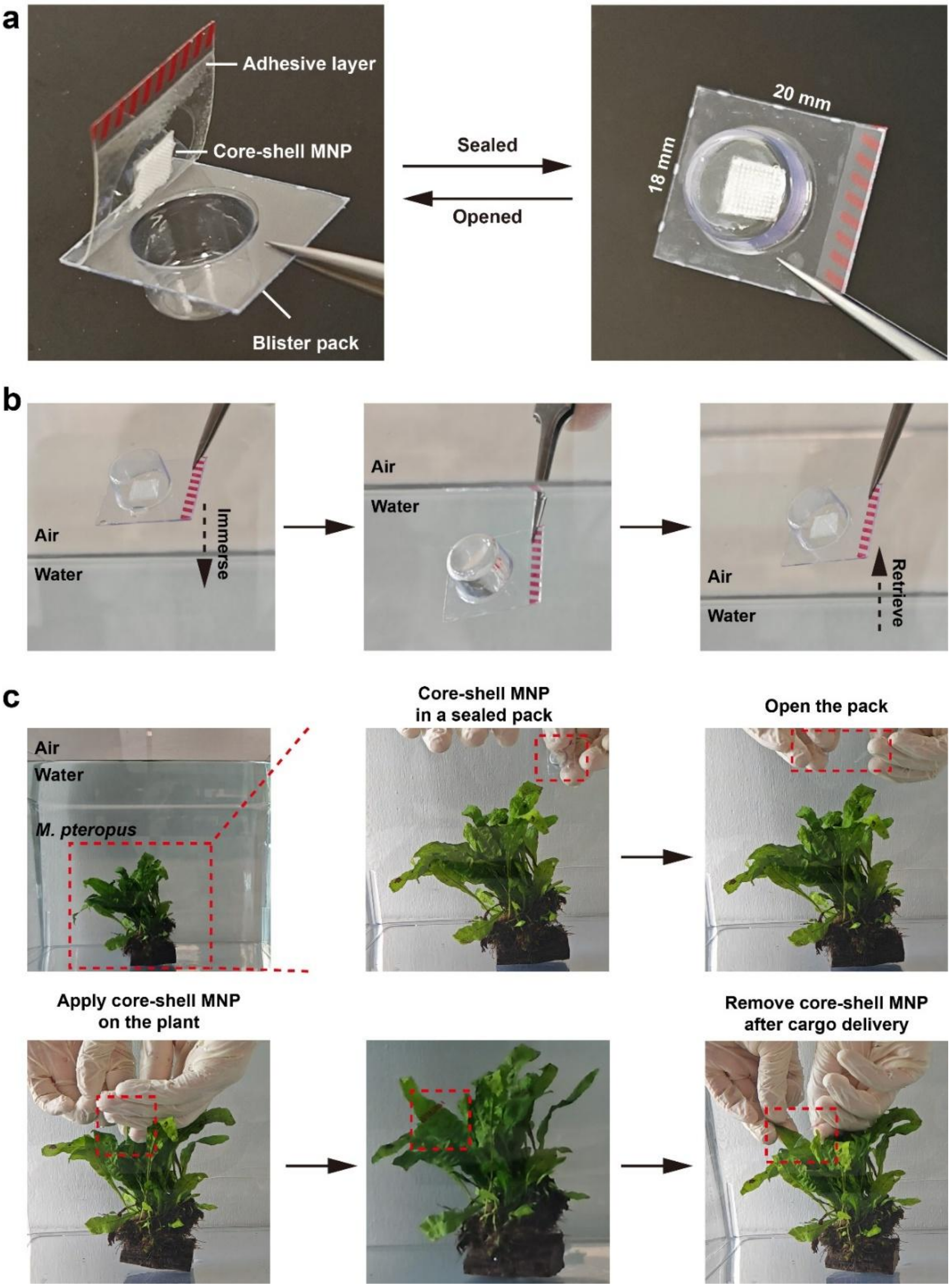

Supplementary Fig. 27. Design of the underwater MNP platform and its application on the aquatic plant. (a) Opened state and sealed state of the underwater MNP platform. (b) Underwater MNP platform was immersed into water and retrieved from water. (c) Photographs illustrating the process to apply the core-shell MNP on *M. pteropus* underwater. Core-shell MNP was initially sealed in the underwater MNP platform before transferring into the water environment. After opening the protective material, the core-shell MNP (stuck on the adhesive layer) was applied on *M. pteropus*. After cargo delivery, the core-shell MNP (with the adhesive layer) was removed from *M. pteropus*.

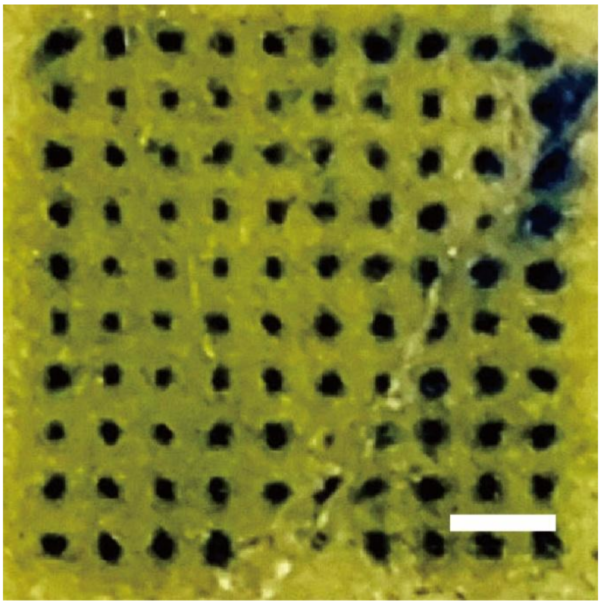

Supplementary Fig. 28. Trypan blue staining image of micropores formed on the leaf surface of *N. benthamiana* after MNP application on the abaxial side. Scale bar, 1 mm.

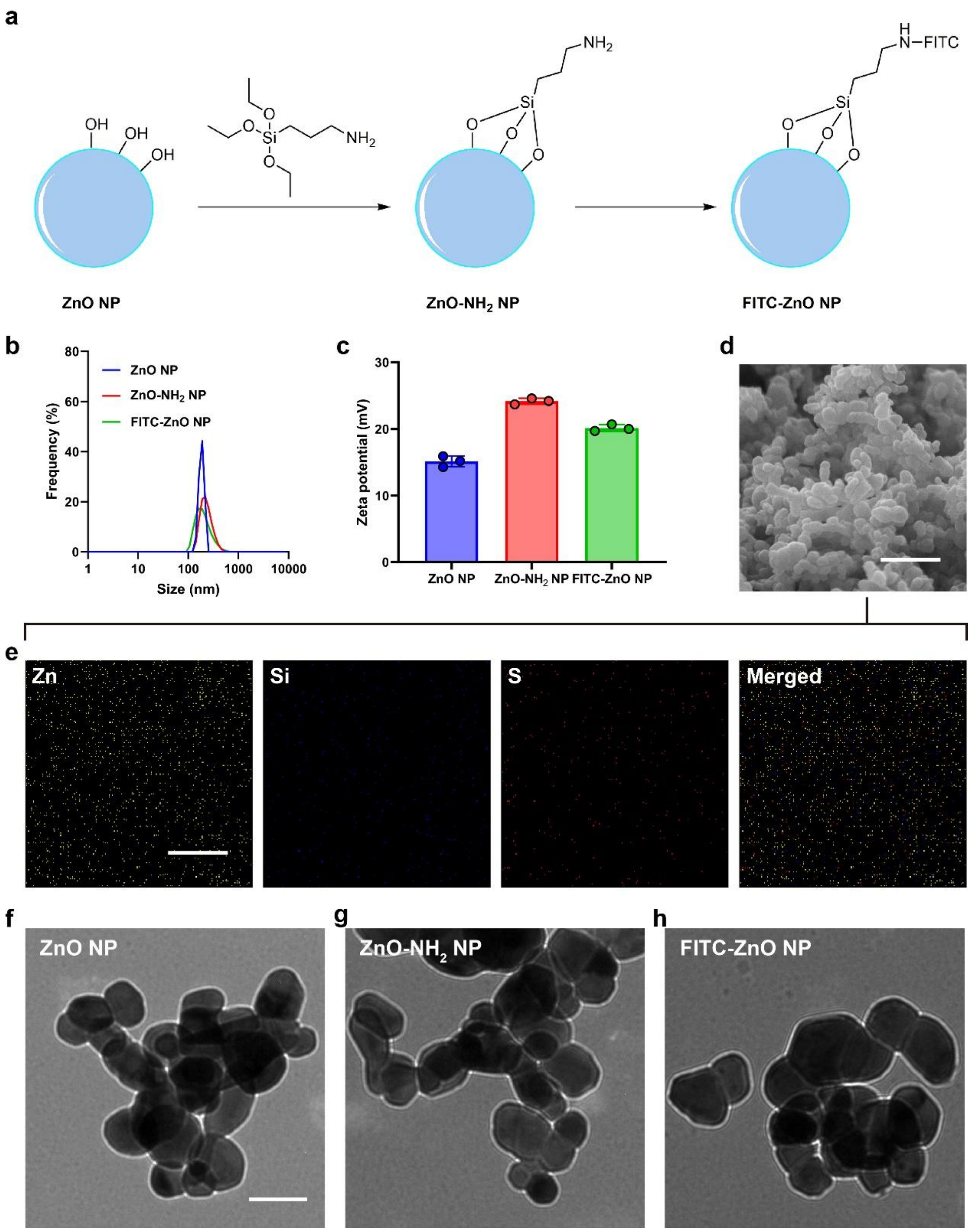

Supplementary Fig. 29. Synthesis and characterization of FITC-ZnO NP. (a) Synthesis of FITC-ZnO NP. (b) DLS data and (c) zeta potential measurement (mean ± s.d., n = 3 independent samples) of different NPs. (d) SEM image and (e) the corresponding EDS elemental mapping results of FITC-ZnO NP (scale bar, 200 μm). TEM images of (f) ZnO NP, (g) ZnO-NH_2 NP and (h) FITC-ZnO NP (scale bar, 100 μm). Source data are provided as a Source Data file.

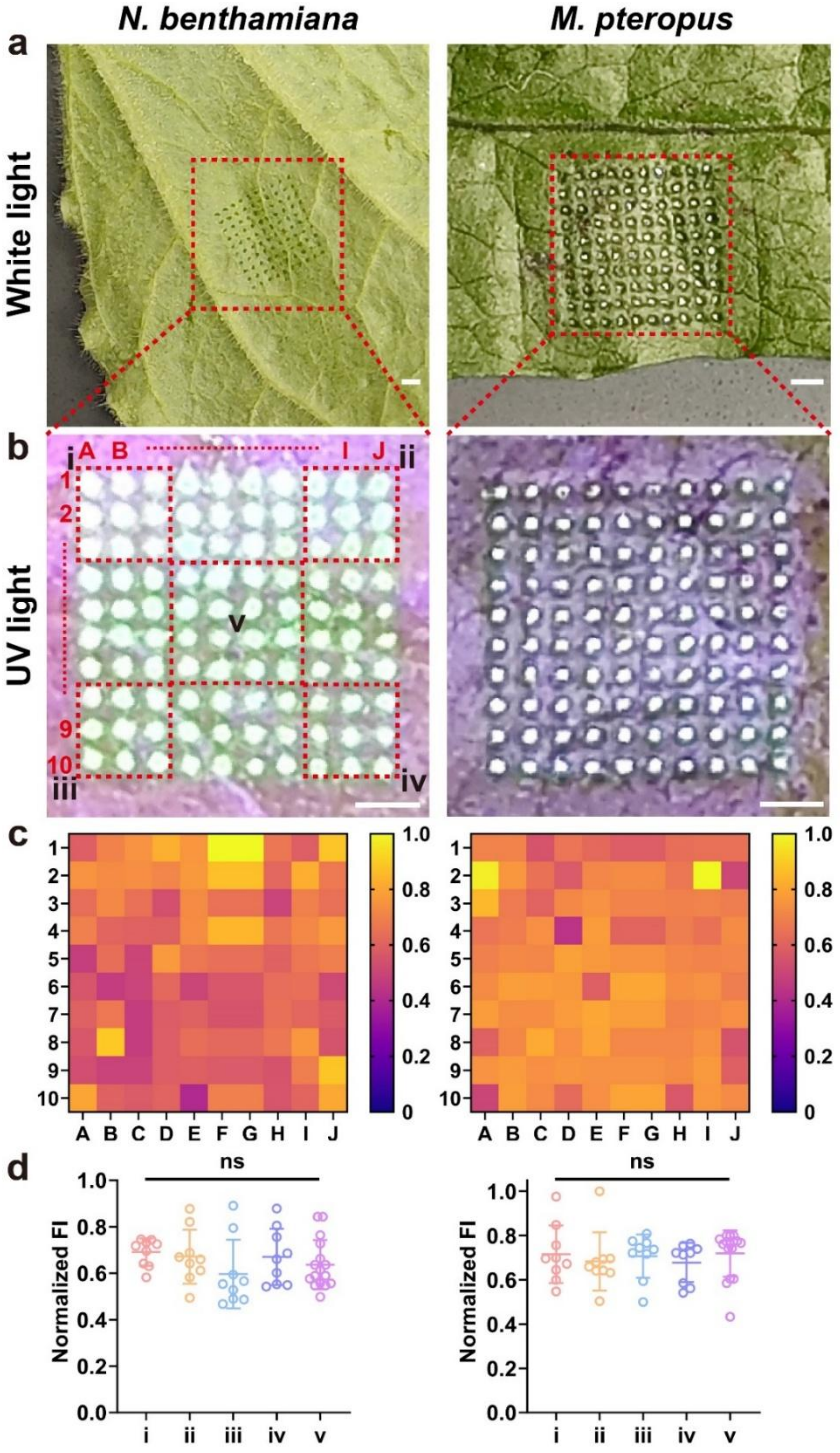

Supplementary Fig. 30. Spatial uniformity of cargo delivery facilitated by core-shell MNPs. (a) Brightfield image and (b) corresponding UV fluorescence image (scale bar, 1 mm). (c) Normalized fluorescence intensity (FI) of NaFI at each micropore across the patch. (d) Comparison of FI among different spatial regions (mean ± s.d., n = 9 technical replicates, i-iv; n = 16 technical replicates, v). Statistical analysis was performed by one-way ANOVA with Tukey's multiple comparisons test. ns $P \geq 0.05$. Source data are provided as a Source Data file.

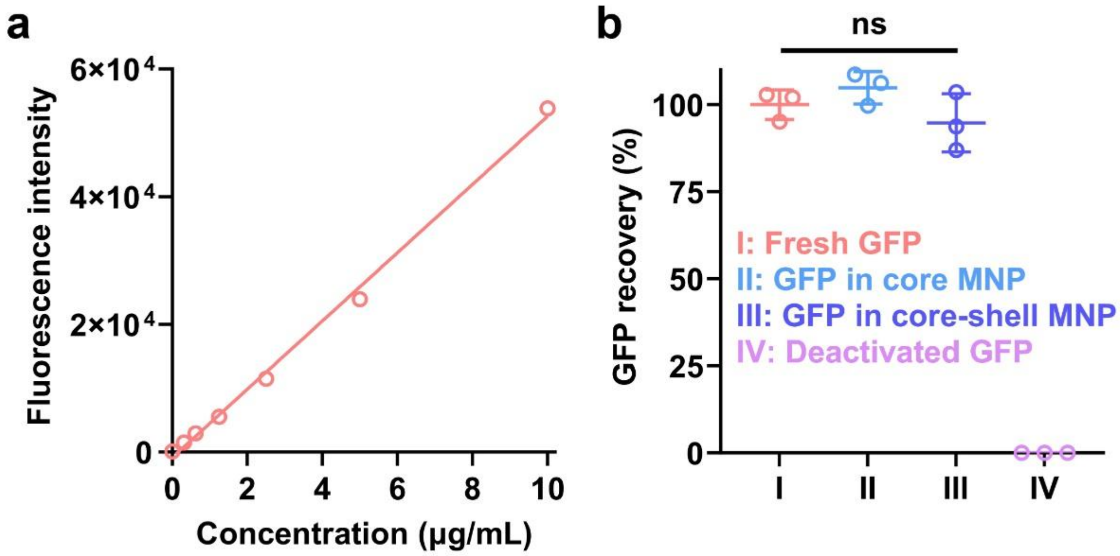

Supplementary Fig. 31. GFP recovery test. (a) Calibration curve of GFP. (b) GFP recovery from freshly-prepared GFP, core MNP, core-shell MNP and denatured GFP (mean ± s.d., n = 3 independent samples). Statistical analysis was performed by one-way ANOVA with Tukey's multiple comparisons test. ns, $P \geq 0.05$. Source data are provided as a Source Data file.

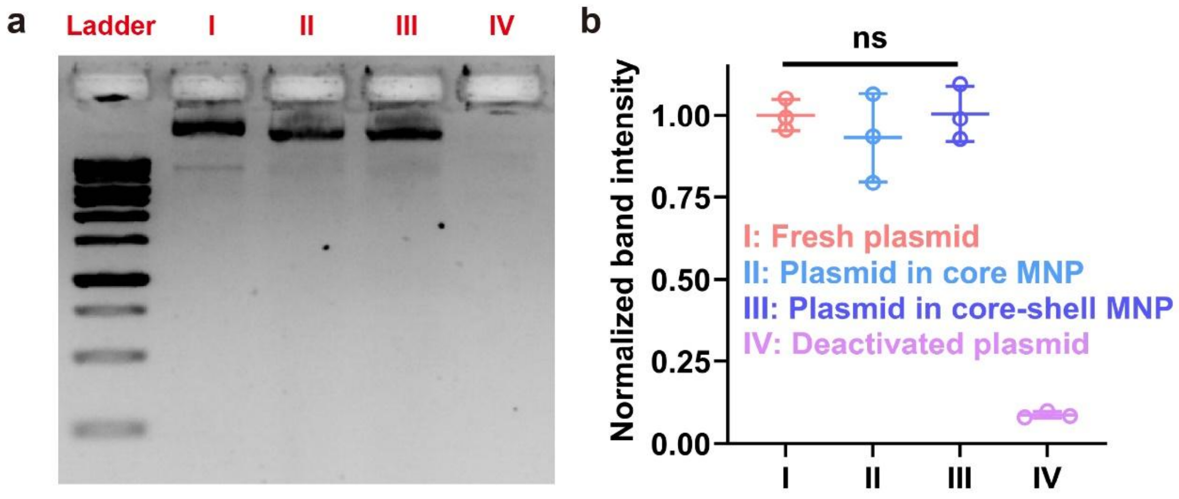

Supplementary Fig. 32. Recovery test of GFP-encoding plasmid DNA. (a) Agarose gel electrophoresis and (b) band-intensity quantification of GFP-encoding plasmid DNA (mean ± s.d., n = 3 independent samples). Statistical analysis was performed by one-way ANOVA with Tukey's multiple comparisons test. ns, $P \geq 0.05$. Source data are provided as a Source Data file.

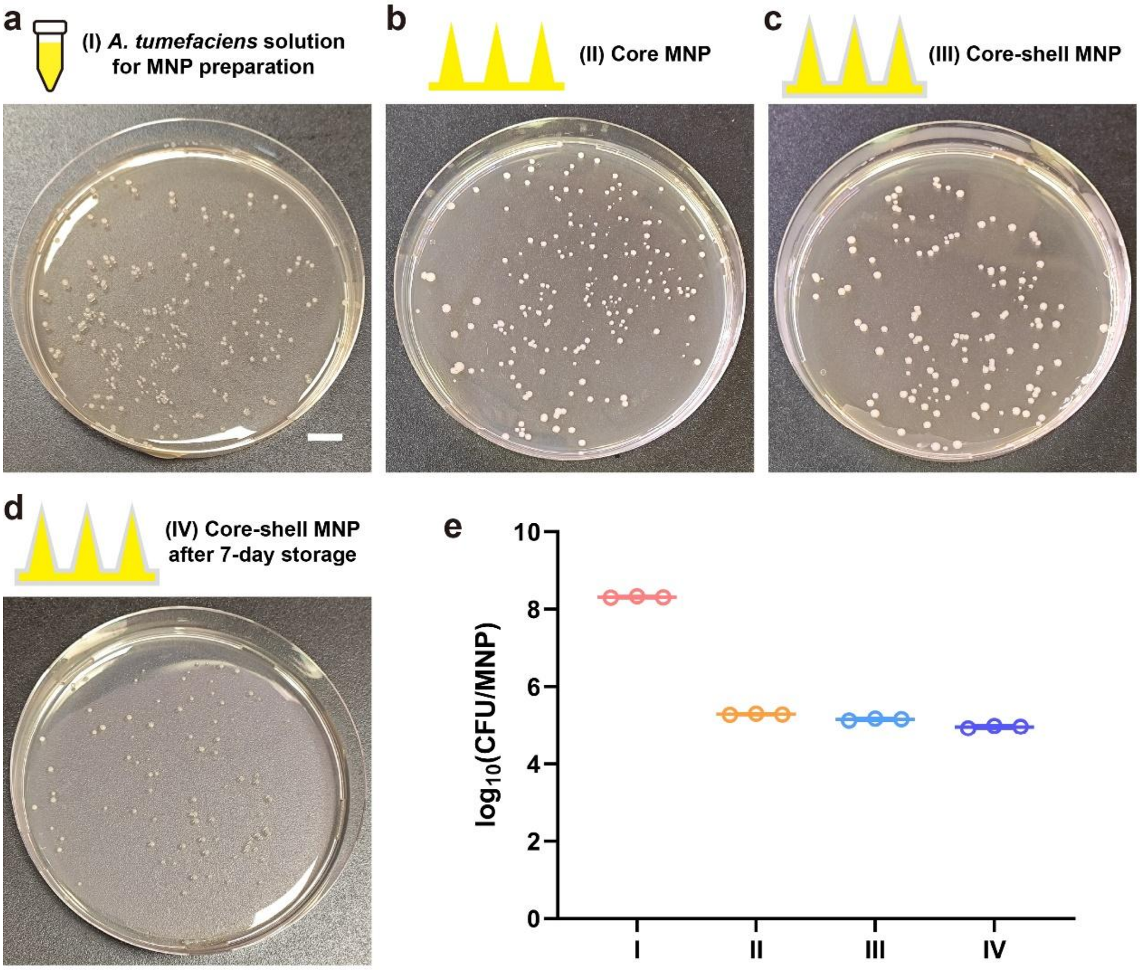

Supplementary Fig. 33. Recovery test of viable *A. tumefaciens*. Bacterial colonies of *A. tumefaciens* from (a) original *A. tumefaciens* solution, (b) *A. tumefaciens*-loaded core MNP, (c) *A. tumefaciens*-loaded core-shell MNP and (d) the core-shell MNP after 7-day storage (scale bar, 1 cm). (e) Quantification of viable *A. tumefaciens* (mean ± s.d., n = 3 independent biological replicates). Quantification was based on a single MNP (b)-(d) or the volume of *A. tumefaciens* solution used to fabricate one MNP (a). Source data are provided as a Source Data file.

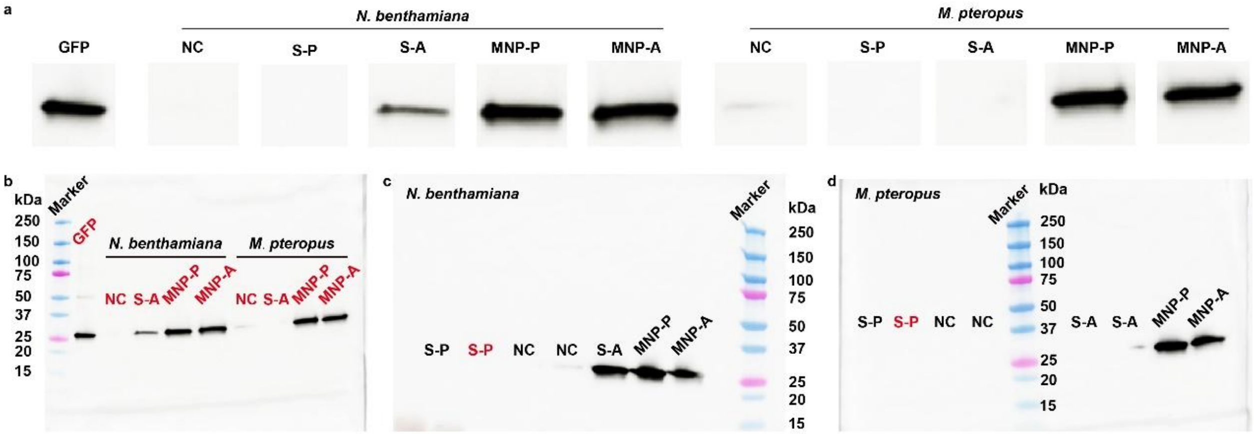

Supplementary Fig. 34. Western blot images of GFP. (a) Representative Western blot images of GFP under different treatment groups. (b)-(d) Raw data of Western blot images after protein extraction from the treated leaves. Panel (a) was collected from the red-labeled lanes of (b)-(d). Source data are provided as a Source Data file.

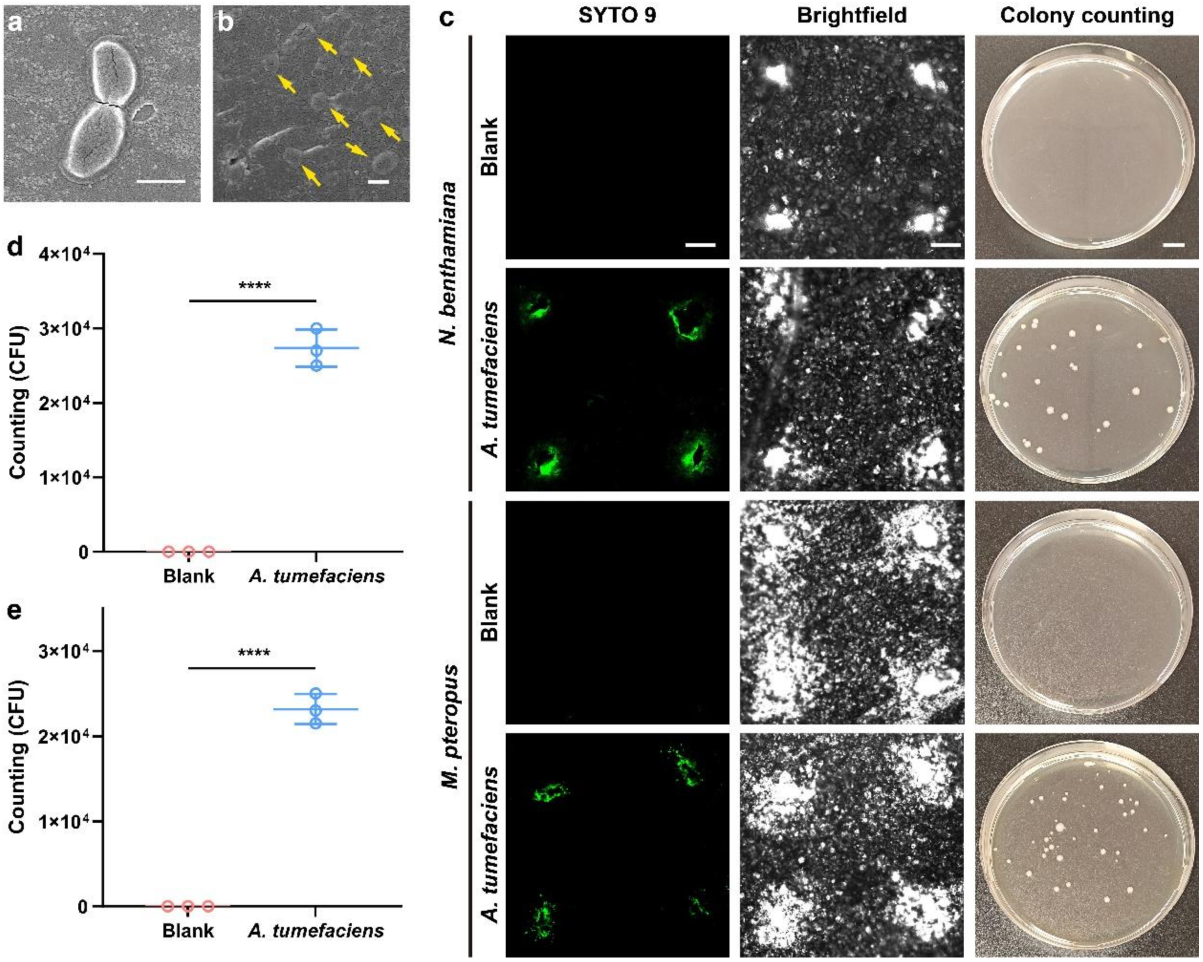

Supplementary Fig. 35. Viable *A. tumefaciens* delivered into plants. (a) SEM image of *A. tumefaciens* (scale bar, 1 μm). (b) Cross-sectional SEM image of *A. tumefaciens* from MNP-A group (scale bar, 1 μm). (c) Confocal images (Ex. 488 nm, Em. 520 nm) of *N. benthamiana* and *M. pteropus* leaves treated by SYTO 9-stained *A. tumefaciens*-loaded core-shell MNP with the counted colonies (scale bar, 100 μm, left; 100 μm, center; 1 cm, right). Quantification of colonies from (d) *N. benthamiana* and (e) *M. pteropus* leaves (mean ± s.d., n = 3 independent biological replicates). Statistical analysis was performed by two-tailed Student's t-test. ****$P < 0.0001$. Source data are provided as a Source Data file.

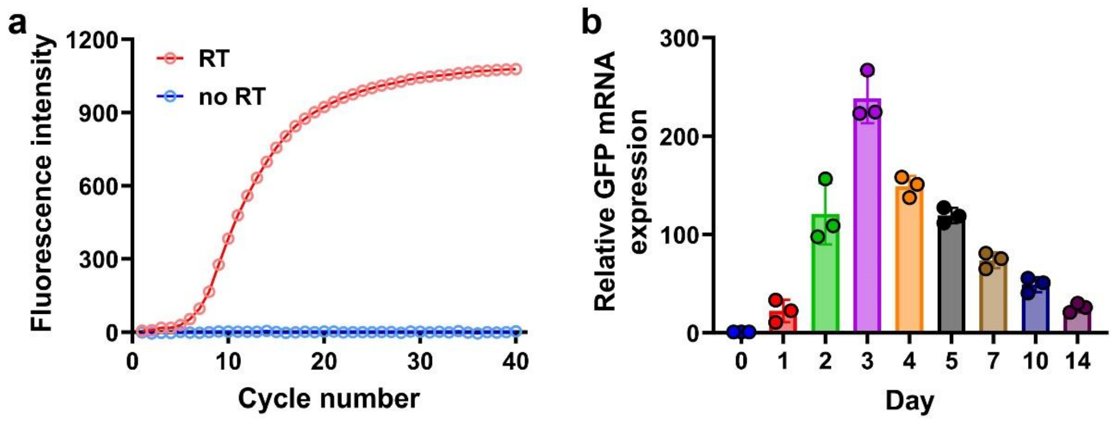

Supplementary Fig. 36. qPCR amplification curves and time profile of GFP mRNA expression. (a) Representative qPCR amplification curves with and without RT from "Day 3" sample of MNP-A in *M. pteropus*. (b) Time profile of GFP mRNA expression for S-A treatment in *N. benthamiana* (mean ± s.d., n = 3 independent biological replicates, each with 3 technical replicates). Source data are provided as a Source Data file.

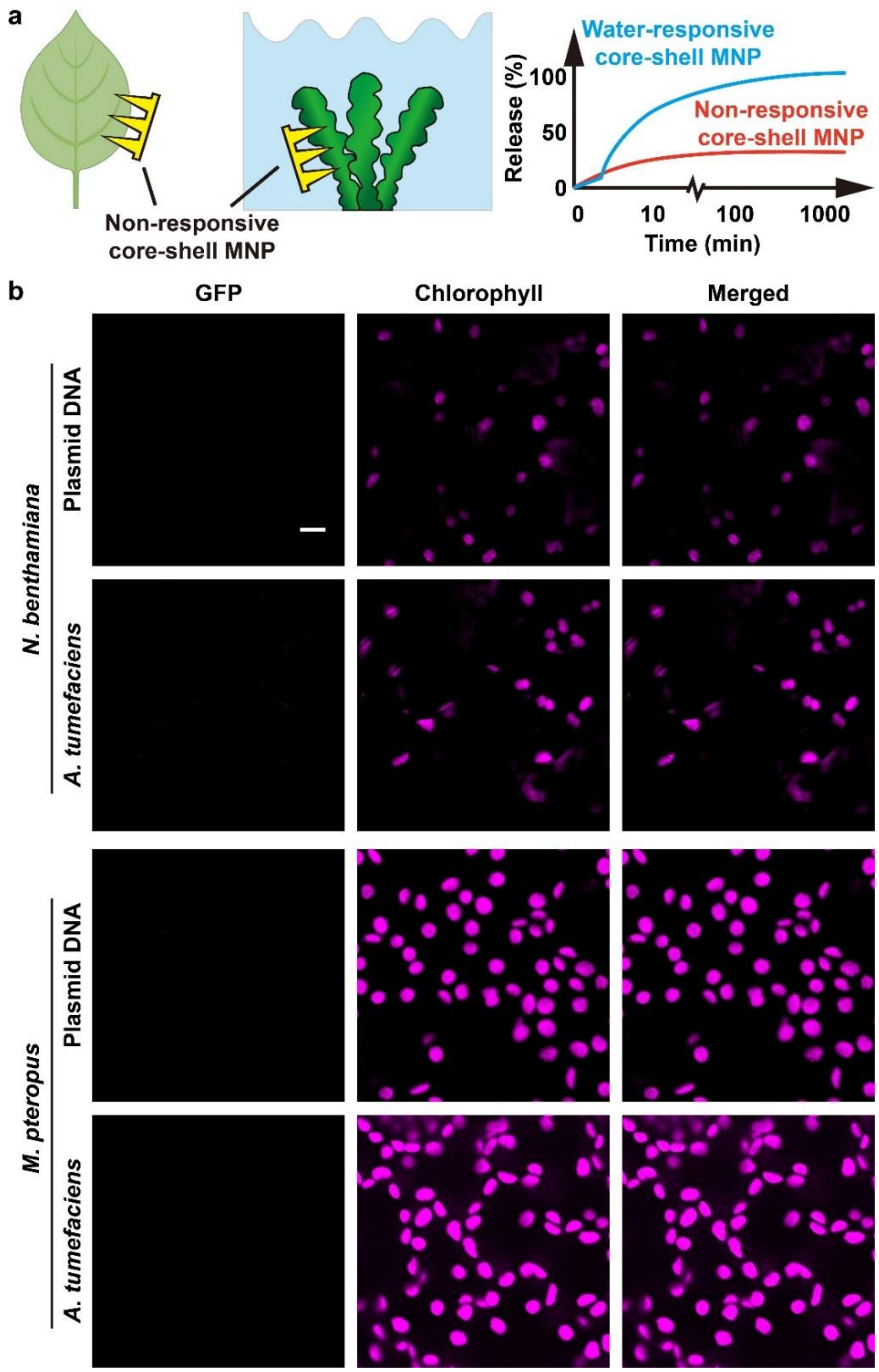

Supplementary Fig. 37. Delivery performance of GFP-encoding DNA or *A. tumefaciens* cargo using non-responsive core-shell MNP. (a) Schematic illustration of non-responsive core-shell MNP applied on terrestrial/aquatic plants, with a schematic comparison of cargo release profiles between the water-responsive and non-responsive core-shell MNP. (b) Confocal images of *N. benthamiana* and *M. pteropus* leaves treated by non-responsive core-shell MNP loaded with GFP-encoding DNA or *A. tumefaciens* cargo (scale bar, 10 μm).

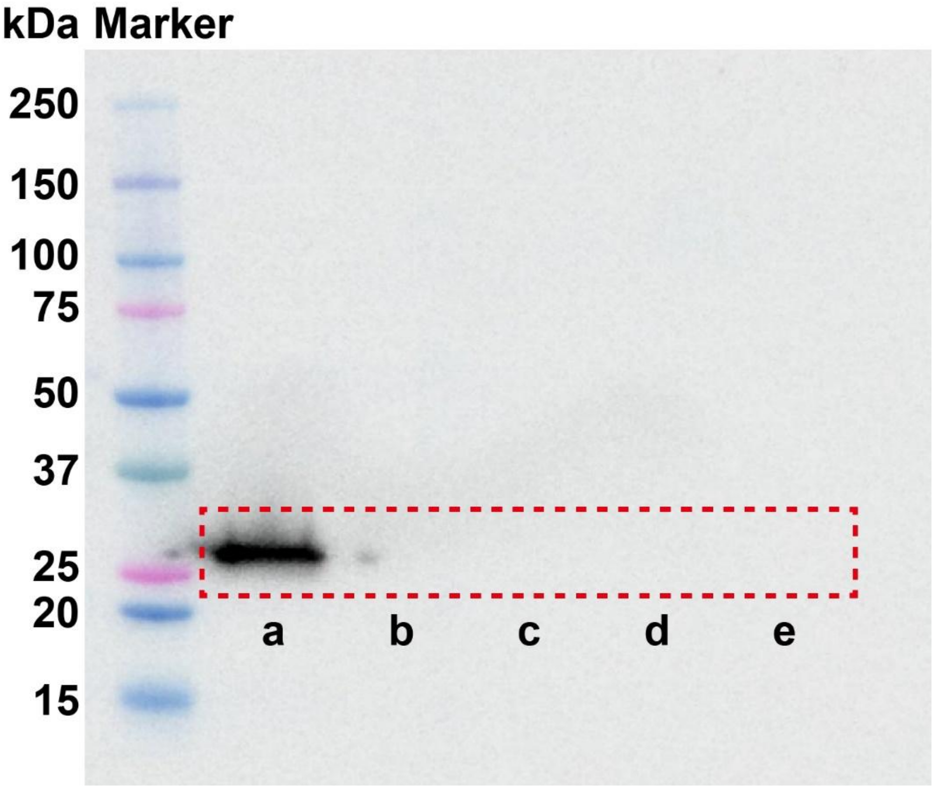

Supplementary Fig. 38. Western blot images of GFP under different treatment groups. (a) S-A of *N. benthamiana*. Non-responsive, core-shell MNP delivery of GFP-encoding (b) plasmid DNA and (c) *A. tumefaciens* into *N. benthamiana*. Non-responsive, core-shell MNP delivery of GFP-encoding (d) plasmid DNA and (e) *A. tumefaciens* into *M. pteropus*. Source data are provided as a Source Data file.

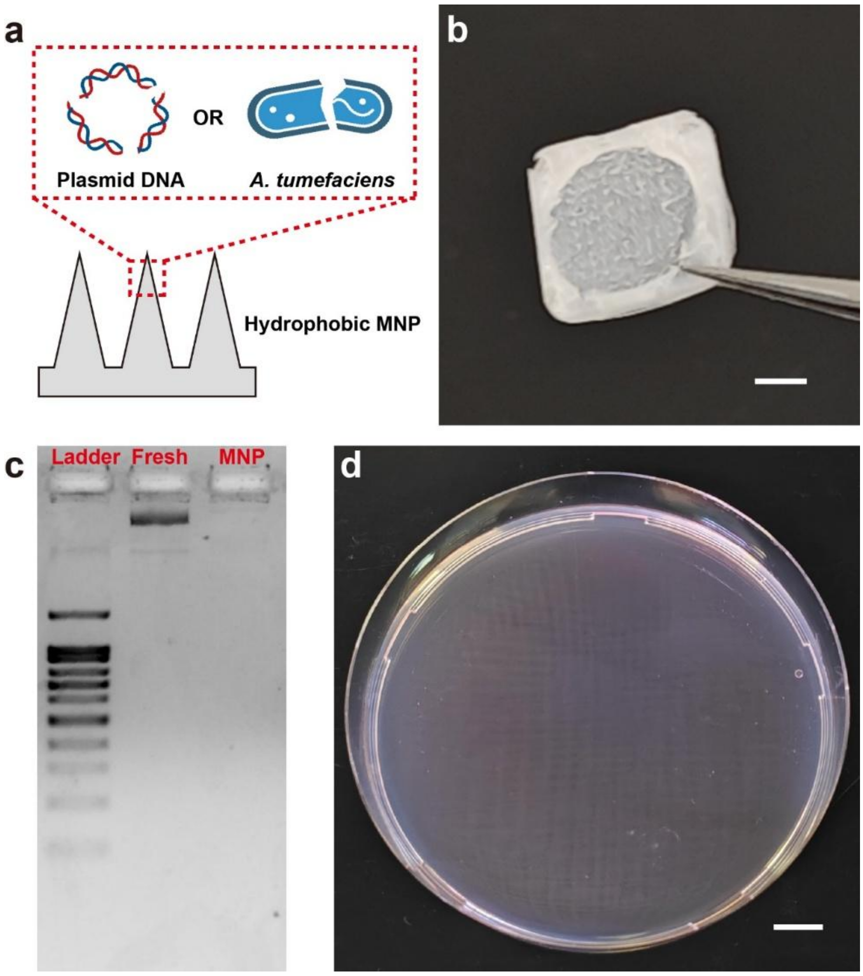

Supplementary Fig. 39. Recovery study of GFP-encoding plasmid DNA and viable *A. tumefaciens* from hydrophobic MNP. (a) Schematic illustration of hydrophobic MNP with the denatured cargo of plasmid DNA or *A. tumefaciens*. (b) Image of the hydrophobic MNP (scale bar, 2 mm). (c) Agarose gel electrophoresis of GFP-encoding plasmid DNA. Lane 1: Ladder. Lane 2: Fresh plasmid DNA. Lane 3: Plasmid DNA extracted from hydrophobic MNP. (d) Image for colony counting of *A. tumefaciens* (scale bar, 1 cm). *A. tumefaciens* was extracted from hydrophobic MNP. Source data are provided as a Source Data file.

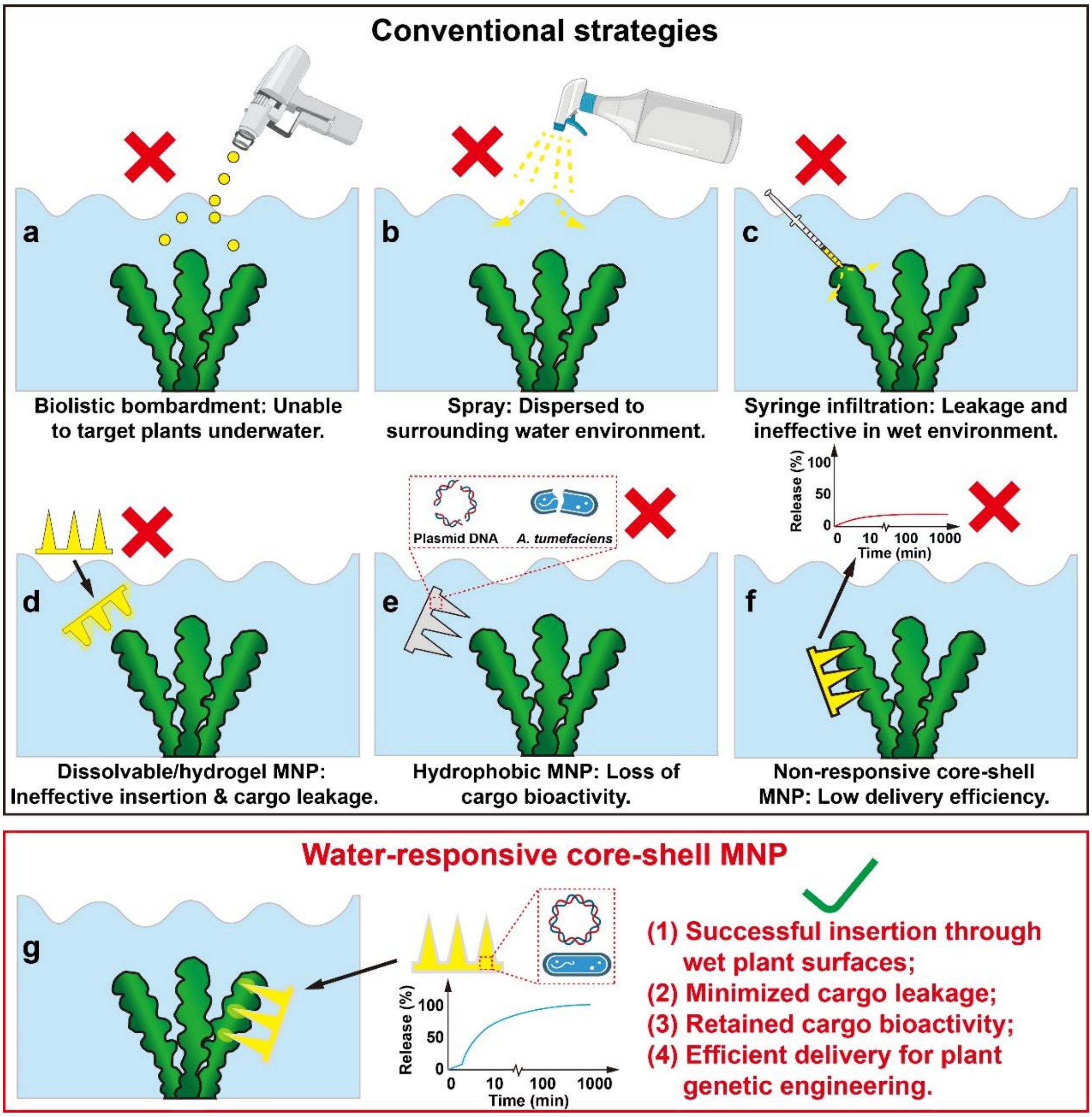

Supplementary Fig. 40. Schematic illustration comparing conventional delivery strategies. (a) Biolistic bombardment, (b) spraying, (c) syringe-mediated infiltration, (d) dissolvable or hydrogel MNP, (e) hydrophobic MNP, (f) non-responsive core-shell MNP and (g) proposed strategy of water-responsive core-shell MNP. Partially created in BioRender. Lew, T.T.S. (2025) https://BioRender.com/6hf5u8f.

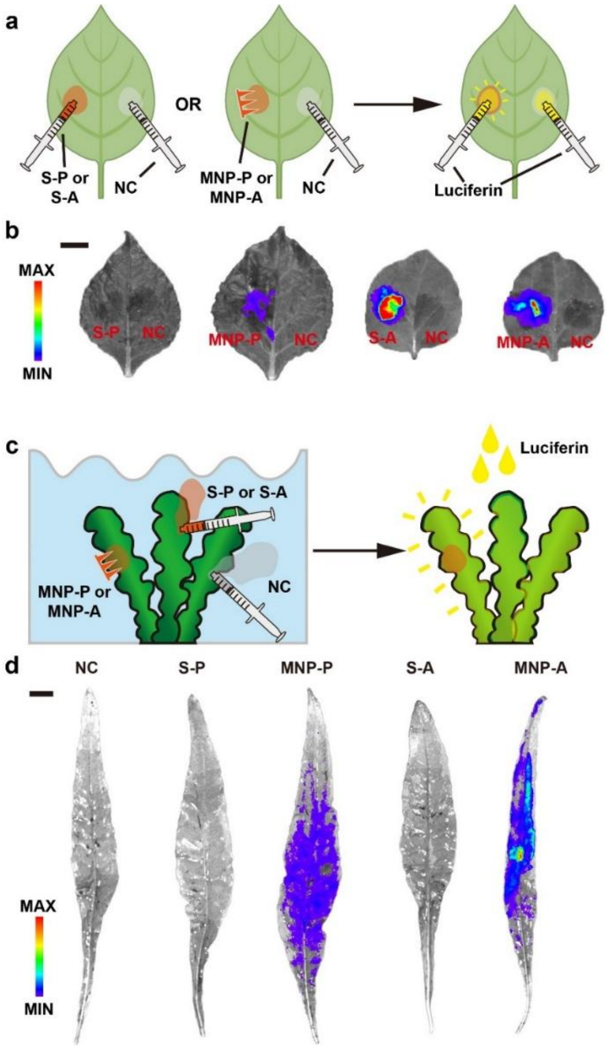

Supplementary Fig. 41. Delivery of firefly luciferase-encoding plasmid DNA and active *A. tumefaciens* by core-shell MNP. (a) Schematic illustration of delivering firefly luciferase-encoding plasmid DNA or *A. tumefaciens* into *N. benthamiana*. Biological cargoes were delivered to the left side of the leaf (S-P, MNP-P, S-A or MNP-A), while buffer infiltration (NC) was performed on the right side of the leaf. (b) Quantitative imaging of chemiluminescence signals in *N. benthamiana* leaves (scale bar, 1 cm). (c) Schematic illustration of delivering firefly-luciferase-encoding plasmid DNA or *A. tumefaciens* into *M. pteropus*. (d) Quantitative imaging of chemiluminescence signals in *M. pteropus* leaves (scale bar, 1 cm). NC: plants treated with infiltration buffer; S-P: plants treated with syringe infiltration of firefly luciferase-encoding plasmid DNA; MNP-P: plants treated with plasmid-loaded core-shell MNP; S-A: plants treated by syringe infiltration of *A. tumefaciens*; MNP-A: plants treated with *A. tumefaciens*-loaded core-shell MNP.

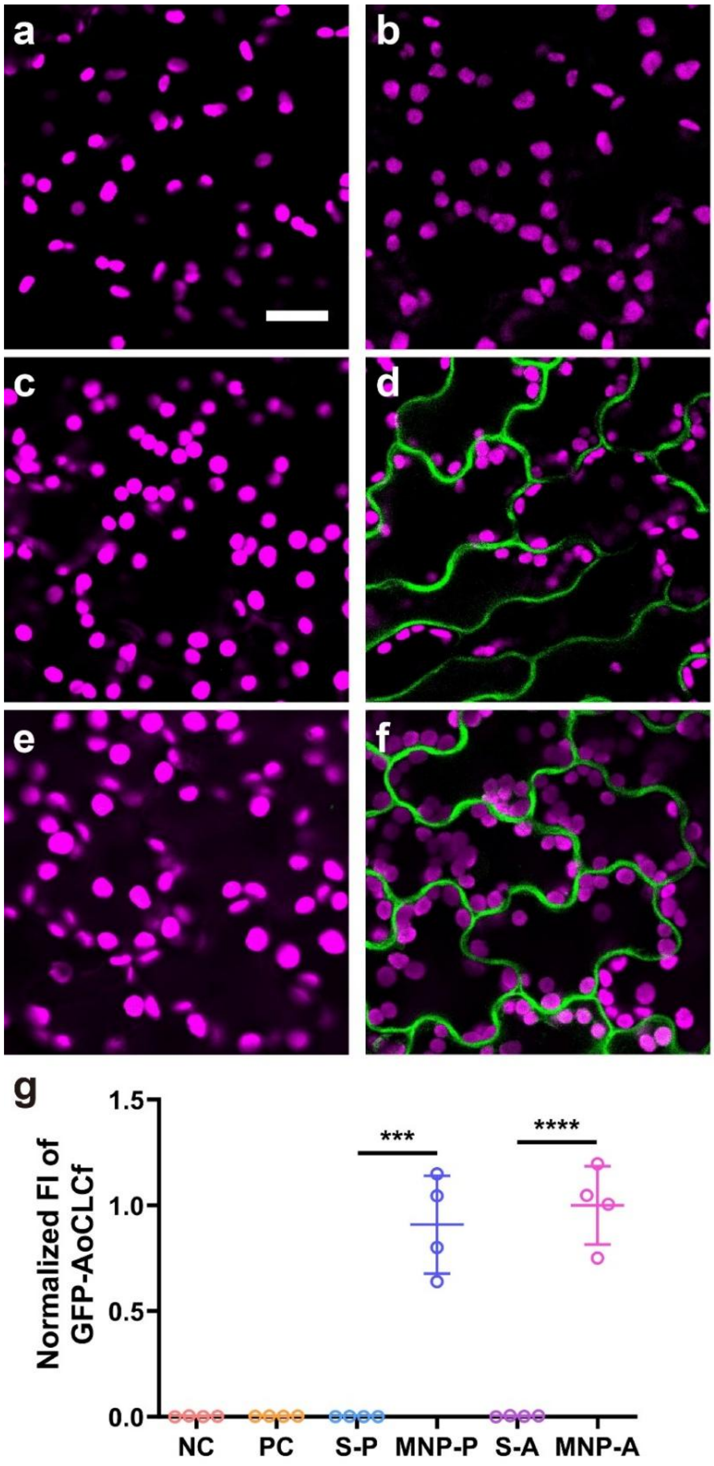

Supplementary Fig. 42. Confocal imaging of GFP-AoCLCf fluorescence in *M. pteropus*. Representative confocal images of (a) NC, (b) PC, (c) S-P, (d) MNP-P, (e) S-A and (f) MNP-A groups. (g) Quantification of GFP-AoCLCf fluorescence intensity (mean ± s.d., n = 4 independent biological replicates). Statistical analysis was performed by one-way ANOVA with Tukey's multiple comparisons test. ***$P < 0.001$ and ****$P < 0.0001$. Source data are provided as a Source Data file.

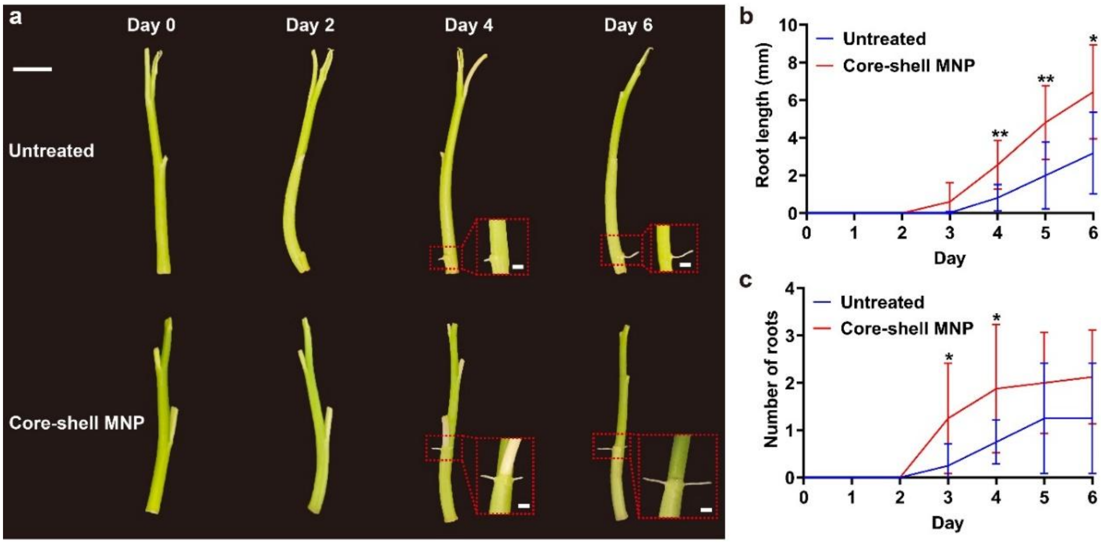

Supplementary Fig. 43. IAA delivery by the core-shell MNP to promote root emergence. (a) Photographs showing root development from *I. aquatica* cut stems. Scale bar, 1 cm, out of the inset; 2 mm, in the inset. (b) Longest root length and (c) number of roots in each stem treatment group. Data of (b) and (c) represent mean ± s.d. (n = 8 independent biological replicates). Statistical analysis was performed by two-tailed Student's t-test. *$P < 0.05$ and **$P < 0.01$. Source data are provided as a Source Data file.

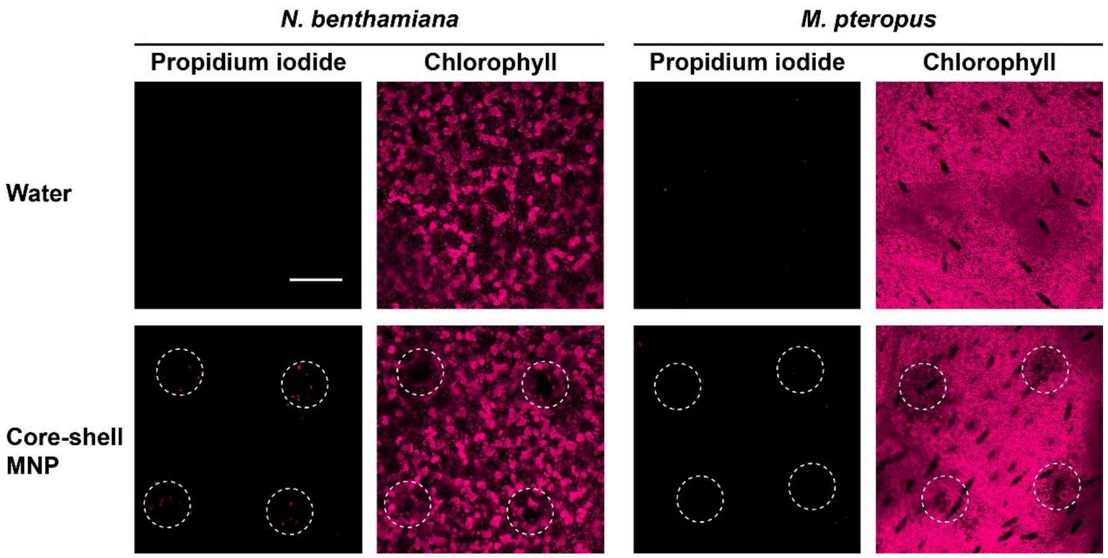

Supplementary Fig. 44. Representative confocal images after PI staining of buffer-treated and core-shell MNP-treated leaves. White dashed circles represented the micropores induced by microneedles. Fluorescence of PI (red, Ex. 488 nm, Em. 570 nm) and chloroplast (magenta, Ex. 488 nm, Em. 670 nm) was presented. Scale bar, 200 μm.

Supplementary Table 1. Representative cargoes with different hydrophobicity used to assess release profiles from microneedles.

Cargo	LogD	Reference
NaFl	0.60	4
RhB	2.34	5
Nile red	3.83	5

Supplementary Table 2. Cost of a blister-pack MNP set.

Material	Cost for each item (USD)	Website
Blister pack	0.0828	https://item.taobao.com/item.htm?ft=t&id=764733236364
Adhesive layer	0.0016	https://item.taobao.com/item.htm?ft=t&id=694567097825
Total	0.0844	

Supplementary Table 3. Primers used in this study.

Primer	Sequence
GFP_F_1	TGGCCCTGTCCTTTTACCAG
GFP_R_1	ATGCCATGTGTAATCCCAGCA
qRTeGFP_F	AGGACGACGGCAACTACAAG
qRTFLeGFP_R	TTCTGCTTGTCGGCCATGAT
MpUBQL_F	CAGCAACGTCTGATTTTTGC
MpUBQL_R	CTCGCTCCTTGATTCTTTCG
NbUBC_FW	AAGCCGGCGACGAACATAAG
NbUBC_RV	CGGAATTGTTGACCTACGAG

Supplementary references

1. Arwani, R. T. *et al.* Stretchable ionic–electronic bilayer hydrogel electronics enable in situ detection of solid-state epidermal biomarkers. *Nat. Mater.* **23**, 1115–1122 (2024).
2. Dimian, A. C., Bildea, C. S. & Kiss, A. A. Chemical Product Design. In *Computer Aided Chemical Engineering* vol. 35 489–523 (Elsevier, 2014).
3. Cai, L., Lu, J., Gao, Z. & Cai, Z. Dynamic analysis model for the diffusion coefficient in high-viscosity polymer solution. *Ind. Eng. Chem. Res.* **57**, 15924–15934 (2018).
4. EMBL-EBI. Fluorescein sodium. (2022) https://www.ebi.ac.uk/chembl/explore/compound/CHEMBL1628233.
5. Alsolmy, E., Abdelwahab, W. M., Martinez, V., Henary, M. & Patonay, G. Investigation of benzophenoxazine derivatives for the detection of latent fingerprints on porous surfaces. *J. Photochem. Photobiol. A: Chem.* **392**, 112416 (2020).
