# Supplementary material for: Amphibious microneedles for programmable delivery of biomolecules and microorganisms in living plants
Source: Nat Commun. 2025 Nov 24;16:10993. doi: 10.1038/s41467-025-66799-1 (PMC12690088; doi:10.1038/s41467-025-66799-1)
Supplement: Supplementary file 2 — Description of Additional Supplementary Files [file 41467_2025_66799_MOESM2_ESM.pdf]

Description of Additional Supplementary Files

File Name: Supplementary Movie 1
Description: Time-lapse mapping of water, K-C10 (aq), and cargo (aq) distributions within a 2×30 µm region, evenly divided into three 2×10 µm sections (water, shell, and core from left to right).

File Name: Supplementary Movie 2
Description: Adhesion of a core-shell MNP in static water.

File Name: Supplementary Movie 3
Description: Adhesion of a core-shell MNP in flowing water.
